# Systematic Review to Update ‘Value of a Statistical Life’ Estimates for Australia

**DOI:** 10.3390/ijerph18116168

**Published:** 2021-06-07

**Authors:** Jaithri Ananthapavan, Marj Moodie, Andrew J. Milat, Rob Carter

**Affiliations:** 1 Deakin Health Economics, School of Health and Social Development, Institute for Health Transformation, Deakin University, Geelong, VIC 3220, Australia; marj.moodie@deakin.edu.au (M.M.); rob.carter@deakin.edu.au (R.C.); 2 Global Obesity Centre, School of Health and Social Development, Institute for Health Transformation, Deakin University, Geelong, VIC 3220, Australia; 3 NSW Ministry of Health, St Leonards, NSW 2065, Australia; Andrew.Milat@health.nsw.gov.au; 4 Sydney Medical School, The University of Sydney, Camperdown, NSW 2050, Australia

**Keywords:** value of a statistical life, cost-benefit analysis, systematic review

## Abstract

The value of a statistical life (VSL) estimates individuals’ willingness to trade wealth for mortality risk reduction. This economic parameter is often a major component of the quantified benefits estimated in the evaluation of government policies related to health and safety. This study reviewed the literature to update the VSL recommended for Australian policy appraisals. A systematic literature review was conducted to capture Australian primary studies and international review papers reporting VSL estimates published from 2007 to January 2019. International estimates were adjusted for income differences and the median VSL estimate was extracted from each review study. VSL estimates were used to calculate the value of a statistical life year. Of the 18 studies that met the inclusion criteria, two studies were primary Australian studies with a weighted mean VSL of A$7.0 million in 2017 values. The median VSL in the review studies was A$7.3 million. For Australian public policy appraisals, we recommend the consideration of a base case VSL for people of all ages and across all risk contexts of A$7.0 million. Sensitivity analyses could use a high value of A$7.3 million and a low value that reflects the value (A$4.3 million) currently recommended by the Australian government.

## 1. Introduction

In order to recommend government action, policy-makers need to weigh up the potential costs and benefits of any proposed policy. Australian governments require cost-benefit analysis (CBA) to assess whether proposed policies represent good value-for-money (see Table S1 for definitions of key terms). The Australian Government mandates that every policy proposal that either recommends the introduction or the abolishment of regulation, must be accompanied by an Australian Government Regulation Impact Statement (RIS) [1]. A key component of a RIS is a CBA. CBA is a type of economic evaluation where both the costs and benefits of a proposed policy action are considered in monetary terms.

The valuation of health improvements is a key variable in economic evaluations of health interventions and policies. There are a variety of different approaches used and there continues to be an active research agenda into methodological developments designed to provide acceptable values for use in economic evaluations to support decision-making [2]. In Australia, economic evaluations in the health sector have predominantly used cost-effectiveness analyses (CEA) and cost-utility analyses (CUA), where the output of the analysis is presented as a cost per unit of health improvement, either in natural units (e.g., cost per ‘case of cancer prevented’) or expressed in quality or disability adjusted life years (cost per QALY or DALY respectively). Both CEA and CUA methods avoid the issues and controversies associated with placing a monetary value on life or health [3]. In Australia, these methods have become the analytical cornerstone of efficiency data to inform decision-making in the health sector, prominently used in the assessment of pharmaceuticals and medical devices [4,5]. There are, however, several situations where health sector evaluations may benefit from the use of a CBA framework.

Firstly, although CEA and CUA do not explicitly place a dollar value on life or health as an input to the analyses, assessment of cost-effectiveness relies on the use of threshold values and therefore the use of these techniques does not negate the need to place a monetary value on health [3]. Secondly, although both CEA and CUA are well placed to aid healthcare budget resource allocation decisions, the results of these analyses preclude the assessment of whether health policies are more or less efficient than interventions in other (non-health) sectors. Although the CBA framework entails unresolved issues related to its underpinning normative assumptions and therefore its use in public policy decision-making [6], according to Dobes [7], CBA is the only viable method that allows comparisons between sectors and therefore is the approach that is best placed to inform resource allocation decisions across sectors. Thirdly, for major health issues such as obesity, effective intervention requires action across several sectors [8,9,10], and therefore harmonisation of methods across sectors may enhance whole-of-government decision-making. On a related point, CBA also allows the capturing of varied effects across sectors in a single evaluation framework [11]. Finally, policies that affect the risk of death and improve health are not limited to the health sector. Many policies in the transport and environment sectors aim to improve safety and health. Therefore using common methods and consistent values in evaluations may result in more consistent decision-making and therefore may increase the efficiency of resource allocation for all health and safety policies across government sectors [3].

The value of a statistical life (VSL) is an estimate of an individual’s willingness to trade their wealth for fatality risk reductions [12]. It is important to note that the VSL is not a price tag on an identifiable life, and does not represent the value governments place on saving identified lives, but the willingness-to-pay for fatality risk reduction of an unidentifiable ‘statistical’ life [13]. The misconception of the VSL terminology is widespread [14] and may contribute to the reluctance to use explicit values in health policy decision-making. There are two key methods for estimating the VSL, namely ‘revealed’ and ‘stated preference’ techniques. Revealed preference (RP) studies infer the value of non-market goods (e.g., safety) from individual behaviour in market situations [12]. RP VSL estimates are often derived from ‘hedonic’ wage studies that use the labour market to estimate the compensating wage differential for a worker accepting a small increase in work related fatality risk [13]. Stated preference (SP) studies use survey methods to elicit willingness-to-pay values for hypothetical scenarios to avoid a small fatality risk. Both methods have proponents and opponents, and pros and cons. RP estimates using hedonic wage studies are preferred by decision-makers in the United States (USA), whilst SP studies are favoured by European agencies [15].

A related concept, the value of a statistical life year (VSLY), is a useful measure in health related evaluations because in many cases health interventions result in a small increase in life years rather than full life expectancy [16]. In addition, the VSLY allows the translation of quality adjusted life years (QALYs) from CUA to a monetary value that can be used in CBA [17]. The preferred methodology to derive a VSLY is from studies eliciting willingness-to-pay for increases in life expectancy [18,19]. However given the limited number of these studies, the current VSLY recommended by the Australian Government is calculated from VSL estimates [16,17]. When VSLY is calculated using VSL studies, it should reflect the average remaining years of life in the study population. The calculation of the VSLY currently recommended by the Australian government assumed that the life saved is of an adult with 40 years of life remaining [16]. The VSLY is a constant, which when summed over 40 years has a discounted value equal to the VSL [17].

When undertaking economic appraisals, it is ideal for the VSL estimate to be specific to the policy being evaluated, however, given the time and expense to conduct primary studies, existing studies are often reviewed to identify estimates that are appropriate to the population, context and type of risk being considered [3,12]. Due to the lack of Australian studies providing robust estimates for policy decision-making, a review of both RP and SP studies was commissioned by the Australian Government in 2007 [20]. Based on this review it was recommended that Australian government policy appraisals use a VSL of A$3.5 million in 2007 values (equivalent to A$4.3 million in 2017 values) and a VSLY of A$151,000 [16] (equivalent to A$186,667 in 2017 values). Policy appraisals in the last 10 years have used this value, inflated over time using the consumer price index [21].

Establishing an appropriate VSL for policy appraisal is an important undertaking, as it is likely to be a major determinant of the benefits estimated for public health and safety policies [18,22,23,24]. The aim of this study is to review the literature in order to update the VSL for use in Australian public policy appraisals. A systematic literature review was conducted to: (i) identify and synthesise the evidence from primary studies estimating the VSL in the Australian setting; and (ii) identify and synthesise the evidence from review papers summarising VSL estimates internationally.

## 2. Methods

### 2.1. Search Strategy and Selection of Studies

VSL is used across several sectors and therefore a broad range of databases were included in the search. Systematic search strategies were designed for JSTOR, Science Direct, ProQuest, SCOPUS, Informit, Embase and relevant EBSCOHost databases (see Table S2 for details of the search strategy across each database). The search was kept broad to capture both Australian primary studies and international review papers. The search terms included permutations of ‘value of life’, ‘value of a statistical life’, ‘value of risk reduction’ and ‘willingness to pay for life, death, fatality, injury or health’. The search was conducted in March 2017 and updated in January 2019. It was limited to papers published in 2007 or later, in order to capture the literature published after the previous review commissioned by the Australian Government (the search period for the previous review was 2005 to June 2007) [20]. The reference lists of the studies included were examined to identify additional relevant publications.

**Figure 1:**
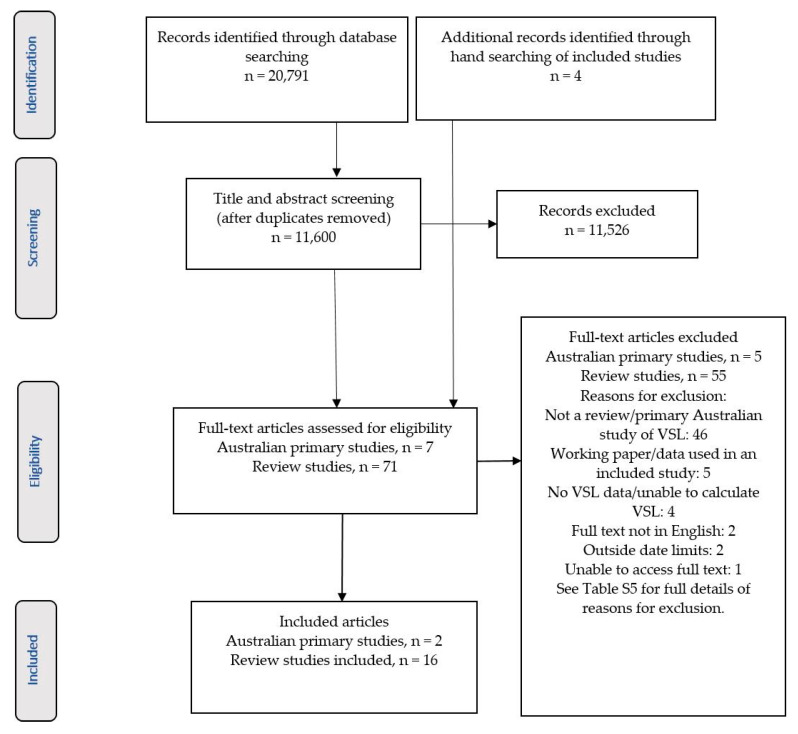
PRISMA flow diagram of article selection process.

Studies were included if they were published between 2007 and January 2019, reported VSL or VSLY estimates from primary studies conducted in Australia, or review papers reporting the synthesis of VSL or VSLY values (not limited to Australian studies). Conference abstracts, newspaper articles and dissertations were excluded. Studies not published in English, or not reporting VSL/VSLY estimates, were also excluded. If studies reported results in both a published article and a working paper or report, only the published paper was included. Once duplicates were removed, title and abstract reviews were conducted to assess eligibility by one reviewer (JA). Full text review was conducted by a single reviewer (JA) and eligibility of studies was presented to co-authors (MM and RC) to achieve agreement on the final list of studies included in the review. A PRISMA flow diagram summarising the study selection process is shown in Figure 1.

### 2.2. Quality Assessment

Despite some guidance on the conduct of SP studies, there are no agreed criteria for the quality assessment of SP studies designed to elicit VSL estimates [25,26,27]. The International Society for Pharmacoeconomics and Outcomes Research (ISPOR) has developed a ‘good practice’ checklist for conjoint analysis (including stated choice experiments) of healthcare studies [26]. Although this checklist is not designed specifically for studies that assess VSL, we have used it to assess whether the primary Australian studies included in this review adhere to good research practice in relation to stated choice methodology (see Table 1). There are no standardised frameworks or guidance for the review of VSL studies. The review studies included were not limited to systematic reviews, and therefore we used a recently developed tool for the quality assessment of narrative reviews (Scale for the Assessment of Narrative Review Articles, SANRA) [28]. Details of the search strategy, data sources and the SANRA score for each of the reviews are presented in Table 1.

**Table 1:** Details of included studies.

Review Studies						
Study	VSL Elicitation Methods and Publication Years of Included Studies	Search Strategy/Data Sources	Country and VSL Context	Number of Studies/VSL Estimates		SANRA Score (Total 12) (See S6) [28]
Anderson & Treich 2008 [29]	RP: 1972–1999; SP: 1982–2005	Not clearly reported—many estimates from a previous review	International; Transport	32 studies; 48 estimates (RP: 12; SP: 36); Results not reported in Table 2 as included studies published prior to 2007		6
Bahamonde-Birke et al., 2015 [30]	RP: 1976–1991; SP (contingent valuation): 1984–1999; SP (stated choice): 2003–2013	RP studies from previous reviews and a literature review of SP studies using stated choice methodology (no search strategy)	International; Transport	50 studies; 66 estimates (RP: 21; SP continent valuation: 32; SP stated choice: 13)		5
Bellavance et al., 2009 [31]	RP: 1974–2004	Key word search of 5 databases and 3 previous literature reviews. Key words not specified	International; Labour market	37 studies; 32 estimates for primary analysis, 39 in sensitivity analysis. Results not reported in Table 2 as included studies published prior to 2007		10
Dekker et al., 2011 [32]	SP (contingent valuation):1983–2008	Key word search using EconLit, Google scholar and EVRI databases	International; Transport; Environment	26 studies; 47 estimates reported (77 estimates from original studies used in the meta-analysis)		10
Doucouliagos et al., 2012 [33]	RP: 1974–2004	Studies included in Bellavance et al. [31]	International; Labour market	See Bellavance et al. [31]; Results not reported in Table 2 as included studies published prior to 2007		9
Hein et al., 2016 [18]	SP: 1996–2011	Key word search using Scopus database	Europe; Environment	7 studies; 22 estimates		10
Hultkrantz & Svensson 2012 [34]	RP: 2005–2009; SP: 1996–2010	EconLit and Pubmed databases and Google Scholar (search strategy not specified)	Sweden; All contexts	12 studies; 48 estimates		10
Lindhjem et al., 2011 [27]	SP: 1970–2008	Search covering academic journal databases and the grey literature	International; Transport; Environment; Health	75 studies; 856 estimates		10
Masterman & Viscusi 2018 [35]	SP: Not reported	Data from 2 studies (Lindhjem et al. [27] and Robinson et al. [22])	International; All contexts	92 studies; 1145 estimates; The results are not reported in Table 2 due to significant overlap between this dataset and other included studies		9
Milligan et al., 2014 [36]	SP: 1970–2013	Uses the SP database reported by Lindhjem et al. [27]. Search strategy for additional studies not specified	International; All contexts	79 studies; 862 estimates. There is significant overlap between this dataset and other included studies. Table 2 reports the results from the three additional studies not included in other included reviews		9
Robinson & Hammitt 2016 [37]	RP: 2003–2014 SP: 2001–2013	Studies from previous reviews and contacts with VSL researchers supplemented with an EconLit database search (strategy not specified)	USA; All contexts	9 studies; 14 estimates		10
Robinson et al., 2019 [22]	RP: 2007–2014 SP: 2003–2014	Studies from previous reviews and contacts with VSL researchers supplemented with a literature search (databases not specified)	International; All contexts	26 studies; 27 estimates (18 SP; 9 RP)		8
Viscusi & Masterman 2017 [38]	RP: 1974–2016	Data from 3 previous studies (Bellavance et al. [31], Viscusi [23] and Viscusi & Aldy [39]) supplemented with a keyword EconLit database search	International; Labour market	68 Studies; 1025 estimates; Table 2 reports estimates from the international studies and a summary of the CFOI dataset		10
Viscusi 2015 [23]	RP: 2003–2014	VSL estimates derived from CFOI data. Search strategy not specified	USA (supplemented with international studies); Labour market	17 studies; 550 estimates. The results are not reported in Table 2 due to significant overlap between this dataset and Viscusi & Masterman 2017 [38]		9
Wheeler & Dockins 2013 [40]	RP: Not reported	RP dataset from the EPA [41]. Search strategy not specified	USA; Labour market	35 studies; 386 estimates		7
Yaduma et al., 2013 [42]	RP & SP: 1974–2009	Data from 2 previous reviews (Viscusi & Aldy [39] and Miller [43]) supplemented with key word search of 3 databases	International; All contexts	83 studies; 83 estimates (21 SP; 62 RP)		10
**Primary Australian Studies**						
**Author**	**Survey Year**	**Population and Risk Context**	**WTP Elicitation Method**	**Mortality Risk Attribute**	**Payment Vehicle**	**ISPOR 10 Item Checklist** [26] **(See S6)**
Hensher et al., 2009 [44]	2007	NSW car drivers *n* = 213; Choice of car based road route based on following attributes: fatality/injury numbers, cost of the trip, travel time, number of speed cameras & speed limit; Public good	Stated choice experiment using face to face interviews to complete a computer survey	Number of deaths per year (range between 0–5)	Car running costs and tolls	Fully compliant on 5 criteria; Mostly compliant on 5 criteria
Hensher et al., 2011 [45]	2007	NSW pedestrians *n* = 99; Choice of pedestrian route based on the following attributes: fatality/injury numbers, cost, travel time, number of lanes of traffic, road speed limit & crossing type (zebra, lights etc.); Public good			Increase in monthly council rates/rent	Fully compliant on 6 criteria; Mostly compliant on 2 criteria; Somewhat compliant on 2 criteria

CFOI: Census of Fatal Occupational Injuries; EPA: Environmental Protection Agency; EVRI: Environmental Valuation Reference Inventory; RP: revealed preference; SANRA: Scale for the Assessment of Narrative Review Articles; SP: stated preference; USA: United States of America; VSL: value of a statistical life. ISPOR: International Society for Pharmacoeconomics and Outcomes Research; *n*: number of participants; NSW: New South Wales.

**Table 2:** VSL values from review papers (A$2017).

Study	Australian Studies Included	Minimum VSL Estimate ^a^	Maximum VSL Estimate ^a^	Median VSL ^a^	Median VSL Estimate Using Income Elasticity of 0.5 ^b^	VSLY Estimate ^a,c^		
						3% Discount Rate	7% Discount Rate	10% Discount Rate
**Stated Preference Studies**								
Anderson & Treich 2008 [29]		All VSL estimates from studies published prior to 2007						
Bahamonde-Birke et al., 2015 [30]-CV studies								
Bahamonde-Birke et al., 2015 [30]–SC studies	Hensher et al., 2011 [45]; Hensher et al., 2009 [44]	3,665,233	116,716,019	7,581,962 ^d^	7,581,962 ^d^	328,014	568,716	775,327
Dekker et al., 2011 [32]		1,704,668	3,344,120	1,878,772	2,088,128	90,337	156,629	213,531
Hein et al., 2016 [18]		-	-	-	-	88,136 (minimum: 37,118; maximum: 468,272) ^e^		
Hultkrantz & Svensson 2012 [34]		2,514,675	24,564,302	7,369,291	8,707,750	376,718	653,161	890,449
Lindhjem et al., 2011 [27]		284,209	10,231,524	4,717,651	6,114,775	264,540	458,664	625,293
Robinson & Hammitt 2016 [37]		9,451,322	15,799,226	15,415,350	13,944,413	603,268	1045,958	1425,948
Robinson et al., 2019 [22]		583,468	28,202,141	11,532,035	2,410,916	104,302	180,841	246,539
Milligan et al., 2014 [36]		1,399,051	17,899,440	15,094,617	16,306,529	705,459	1,223,139	1,667,496
Median SP VSL		1,399,051	17,899,440	7,581,962	7,581,962	328,014	568,716	775,327
Mean SP VSL		2,755,655	30,965,253	9,084,240	8,370,241	353,234	612,444	834,940
**Revealed Preference Studies**								
Anderson & Treich 2008 [29]		All VSL estimates from RP studies published prior to 2007						
Bahamonde-Birke et al., 2015 [30]-CV	Kniesner & Leeth 1991 [46]							
Bellavance et al., 2009 [31]	Kniesner & Leeth 1991 [46] Miller et al., 1997 [47]							
Doucouliagos et al., 2012 [33]								
Hultkrantz & Svensson 2012 [34]		4,049,761	7,043,063	5,546,412	6,026,316	260,713	452,029	616,248
Robinson & Hammitt 2016 [37]		3,808,498	37,722,267	15,596,707	14,108,465	610,366	1,058,264	1442,723
Robinson et al., 2019 [22]		1,642,971	111,900,063	17,101,389	7,014,189	303,450	526,128	717,267
Viscusi & Masterman 2017 [38] ^f^	Kniesner & Leeth 1991 [46] Miller et al., 1997 [47]	189,912	20,074,339	3,536,095	3,715,149	160,726	278,670	379,909
				17,964,045	17,399,366	752,738	1,305,111	1799,249
Wheeler & Dockins 2013 [40] ^g^			10,842,282	8,229,684	10,757,518	465,396	806,912	1,100,058
Median RP VSL		2,725,735	20,074,339	11,913,195	8,885,852	384,423	666,520	908,662
Mean RP VSL		2,422,786	37,516,403	11,329,055	9,836,834	425,565	737,852	1,005,909
Yaduma et al., 2013 [42]	Kniesner & Leeth 1991 [46] Miller et al., 1997 [47]	990,572	8,464,034	2,261,696	1,007,897	43,604	75,601	103,067
Median all studies VSL		1,521,011	17,899,440	7,905,823	7,298,074	315,732	547,422	746,297
Mean all studies VSL		2,497,608	31,754,063	9,603,764	8,370,241	362,117	627,845	855,936

^a^ International studies have been translated to Australian values by adjusting for income using The World Bank Gross National Index (GNI) values [48], converted to A$ using OECD purchasing power parities (PPP) [49] and inflated to 2017 values using the gross domestic product price deflator index values [50]; ^b^ The impact of the income elasticity varies depending on the income of the country the median estimate is from. If the estimate used is from a country with a higher income compared to Australia, an income elasticity below 1 results in a higher estimate and an elasticity over 1 results in a lower estimate. The opposite occurs if the estimates used is from a country with a lower income to Australia; ^c^ The VSLY is calculated using the median estimate, assuming an income elasticity of 0.5; ^d^ The median estimate is from an Australian study and therefore various income elasticities are not relevant; ^e^ This study reports VSLY. Therefore the discount rates are not relevant. The minimum and maximum values are reported. The median value from the study is used for the summary calculations; ^f^ The first row represents values from international studies. The second row presents values used to estimate the VSL using Census of Fatal Occupational Injuries (CFOI) data. Primary data used to calculate the VSL was not reported. The median value for this CFOI dataset is reported here; ^g^ The maximum value is the mean VSL of the sample. The median value is the publication bias corrected estimate; A$: Australian dollars; CFOI: Census of Fatal Occupational Injuries; CV: contingent valuation; GNI: Gross National Income; OECD: Organisation for Economic Co-operation and Development; RP: revealed preference; SC: stated choice; VSL: value of a statistical life; VSLY: value of a statistical life year.

### 2.3. Data Extraction and Analysis

Separate standardised data extraction forms were developed to collect relevant data from the primary Australian VSL studies, and the review VSL studies. For the primary studies, information on study setting/context, population, survey design features, VSL/VSLY values recommended and the range of values reported was extracted. For the review papers, data on search strategy and data sources used to identify VSL estimates, risk context, VSL elicitation methods, publication years for the included studies, details of any Australian studies included, and the range of VSL estimates were extracted.

Meta-analysis can be used to synthesise data from multiple studies, however in the case of the VSL, it has been suggested that whilst these methods (particularly meta-regression) are suitable to identify factors that influence the VSL estimate, given the various methods and contexts from which the VSL is estimated, meta-analyses should not be used to determine the most appropriate value for use in decision-making [27,41]. Studies have also found that VSL estimates are skewed to the right towards higher values [27], and therefore median values provide more valid estimates of central tendencies than mean values. For the review papers, the VSL for each of the included studies published from 2007 onwards was extracted to determine the minimum, maximum and median value in each review. To avoid duplication of data caused by several reviews reporting on the same primary study, data from original studies were only included once. Review papers with all included studies published prior to 2007 were included in the narrative review, but VSL data were not extracted. For the primary studies, when median values were not available (e.g., when two estimates were provided), the mean value was extracted.

### 2.4. VSLY Calculation

The median VSL estimate in each of the included review papers was used to calculate the VSLY. To align with the methods used to calculate the VSLY currently recommended by the Australian Government for decision-making, a 40 year discount period and a 3% discount rate were used [16]. However it is also recommended that the discount rate used to calculate the VSLY should be the same as the overall discount rate used in the CBA to maintain methodological consistency [51]. Currently, the Australian government recommends a discount rate of 7%, with 3% and 10% used in sensitivity analyses [52]. Therefore VSLY estimates are presented using discount rates of 3%, 7% and 10% (see S3 Equation (1) for VSLY calculation).

### 2.5. Value Transfer Calculation

Income is a key determinant of VSL [42]; therefore to estimate the VSL for Australia from international values, the income differences need to be accounted for, as well as the elasticity of willingness-to-pay for reductions in mortality risk relative to income. Australian VSL estimates were calculated using Gross National Income (GNI) [48,53] per capita data (for Australia and the VSL country of origin for a specific year). There is a lack of consistency in the literature on the VSL income elasticity, however most studies estimate elasticities between 0.3 and 0.8 for high income countries and between one and 1.2 for lower income countries [22,27,31,35,54,55]. For the base case we have used an elasticity of 0.5, however given the lack of agreement we have also presented results using a range of elasticity values from 0.3 to 1.2 (see S3 Equation (2) and Table S4). All VSL and VSLY estimates have been converted to Australian dollars using purchasing power (PPP) parities [49] for the year of the estimate and adjusted to 2017 values using Australian Gross Domestic Product implicit price deflators [50,56].

## 3. Results

After duplicates were removed, the search identified 11,600 records that were screened for inclusion. Full text review was undertaken for 74 records, resulting in two primary Australian studies and 15 review articles meeting inclusion criteria. Hand searching of the reference lists of included studies resulted in four additional review studies being found, for which full text review was completed, with one study meeting the eligibility criteria and being included in the review (Figure 1). Table S5 provides details of the reason for exclusion after full text review.

Details of the studies included are provided in Table 1. The primary aim of many of the included review papers was to investigate theoretical and empirical aspects of VSL estimates rather than to review the range of VSL values in the literature. The SANRA score for the review component of these studies ranged from five to 10 out of a total score of 12, with a mean score of 8.9. Although there aren’t cut offs for different grades of quality, it is suggested that a score of four or lower represents poor quality [28]; all of the studies included in this review scored above this threshold (see S6 for details of quality assessment). The majority of the studies reported VSL estimates from several countries [22,27,29,30,31,32,33,35,36,38,42], with three studies limiting estimates to the USA [23,37,40], one to European estimates [18], and one to estimates derived from the Swedish population [34]. None of the review studies focused on the Australian population. There was an even mix of review papers that reported VSL estimates from RP studies [23,31,33,38,40], SP studies [18,27,32,36], or a combination of both [22,29,30,34,37,42]. Eight studies reported VSL estimates from various contexts [22,27,32,34,35,36,37,42], five were limited to hedonic wage studies [23,31,33,38,40], two reported only transport scenarios [29,30] and one reviewed VSL estimates from environmental hazards [18].

The two Australian studies used ‘stated choice’ experiments to estimate the VSL in a transport context for car drivers [44] and pedestrians [45]. Both studies were undertaken by the same authors, shared similar design features, and were undertaken in 2007, targeting the urban and rural New South Wales population. Both studies either fully or partially met all 10 ISPOR good research practice for conjoint analysis checklist criteria (see S6). Although these studies are SP studies, they differed in their methodology compared with the majority of the SP studies included in the review papers, which used ‘contingent valuation’ methods to estimate VSL.

The reported VSL values varied dramatically, both within each of the review studies and between studies. The minimum VSL was A$189,912 [38] and the maximum value was over A$116 million [30] (see Table 2). When looking at the median values calculated using an income elasticity of 0.5, the VSL estimates reported in each of the review studies, ranged from approximately A$1.0 million [42] up to over A$17 million [38]. SP studies generally reported lower VSL estimates compared to RP studies. The median value from VSL review studies (calculated using an income elasticity of 0.5) reporting RP estimates was approximately A$8.9 million, whereas the corresponding value for SP studies was approximately A$7.6 million.

The majority of the estimates reported in the review papers were from countries with marginally different income levels to Australia, and therefore the income elasticity chosen had a relatively small impact on the VSL estimates. However when the median estimate was from a lower income country, the income elasticity had a drastic impact on the results [22].

The median VSLY calculated using a 3% discount rate from the median SP VSL estimates using an income elasticity of 0.5 was A$328,014. The corresponding value from RP studies was A$384,423. The study by Hein and colleagues [18] was the only study that aimed to review estimates of VSLY from SP studies. The median estimate from this study was A$88,136, which is over 70% lower than the median estimate calculated from the median SP VSL estimates.

The mean VSL (weighted by the sample size) from the five estimates reported in the two primary Australian studies was A$7.0 million (see Table 3). The corresponding VSLY estimates were A$303,531, A$526,269;and A$717,458, using a 3%, 7% and 10% discount rate respectively. These values are similar to the central VSL and VSLY estimates from the review studies.

## 4. Discussion

Regulatory agencies around the world develop recommendations on the appropriate VSL for use in social CBA by reviewing the literature [22,41]. Here, we have reviewed both the international literature and local evidence to make recommendations on the range of VSL values that could be considered for use in Australian CBA. Some authors have recommended that international VSL estimates can be translated to other countries by adjusting for differences in income and income elasticities [22,36,38]. However this approach would ignore empirical evidence that governments outside the USA use VSL values that are lower than USA estimates, even after adjustment for income differences [38]. Whilst differences in recommended VSL values across different countries may be partly due to the preference for different study methodologies in different jurisdictions [15], there may also be important cultural, social, institutional and other factors that influence the VSL [22,57]. Therefore, estimates based on Australian cultural norms reflected in preferences of the Australian population have greater relevance for Australian government decision-making. It is important to acknowledge, however, that contemporary Australian evidence is limited in context and not necessarily representative of the Australian population, as the two studies cited were limited to the transport context and to the New South Wales population [44,45]. Nonetheless, we place emphasis on the importance of the most up to date Australian preferences and recommend the consideration of a new VSL value of A$7.0 million for public policy decision-making, based on the most recent Australian studies. This aligns well with the median VSL estimates from all of the international studies. There are two older Australian RP estimates of VSL based on hedonic wage studies conducted using data from 1984/85 [46] and 1992/93 [47] that were not included in our review (due to date limits for the search). These estimates are higher than the estimates from the contemporary Australian studies and vary between A$9.4 million and A$34.5 million in 2017 values. These studies are limited by small samples sizes, and the poor quality of the mortality risk data, which is a key variable in the calculation of VSL using hedonic wage models [38].

Given the wide range of VSL estimates in the literature and the limited Australian evidence, uncertainty remains around the VSL and VSLY estimate most appropriate for the Australian setting. It is therefore essential that sensitivity analyses are conducted with high and low values. However, given that the value of fatality risk reduction is a major benefit for many public health and safety interventions, using a wide range of VSL values in the evaluation may result in the CBA being indeterminate [58]. Therefore, sensitivity analyses should be implemented in a way whereby confusion and likelihood of decision paralysis is minimised. This involves using techniques that allow uncertainty to be transparent such as probabilistic analyses where variations in input parameters relate to the probability of various outcomes. For univariate sensitivity analyses, we recommend the consideration of a high VSL based on the median estimate from all international studies assuming an income elasticity of 0.5 (A$7.3 million) and a low estimate based on current Australian guidance inflated to 2017 values (A$4.3 million).

To place the recommended VSL value from this review in the international context, we compare it to the VSL used in policy decision-making in two countries similar to Australia in terms of socioeconomic factors and system of government: New Zealand and Canada [59]. The VSL used in New Zealand policy decisions in the transport sector is based on a New Zealand SP study conducted in 1991 and indexed to the year of policy analysis (A$5.6 million in 2017 values) [60]. The Canadian VSL used for policy decision-making is based on a review commissioned by the Canadian Government in 2009 of Canadian VSL studies. The central value is A$7.0 million in 2017 values with a high value of A$10.3 million and a low value of A$3.8 million [61]. These results provide confidence in our recommendations for several reasons: firstly, the central VSL used in Canada and New Zealand are comparable to our recommendation; and secondly, both countries have shown a preference for using values elicited from their own populations, despite the New Zealand value being based on a single study conducted over 25 years ago. It is reported that a new SP study of the New Zealand population and an updated review of Canadian VSL evidence will be undertaken to update the VSL for decision-making [60,62].

There remain several unresolved issues related to VSL estimations including: (i) whether the VSL should be adjusted for the cause of death; (ii) whether the VSL from different risk contexts should be varied; and (iii) whether the VSL varies with age and the related issue of applying a constant VSLY. There are also several unresolved issues related to the methods for estimating VSL and the VSLY. A discussion of each of these follows.

Efficiency in public policy decision-making is enhanced by the incorporation of societal preferences [30]. It follows, therefore, that if there is evidence of preferences for varied VSL values for different risk contexts, these differences should be reflected in recommended VSL values. It is feasible that willingness-to-pay for risk reductions could vary based on the source of risk, whether there is suffering involved, the degree of control over the risk and the timing of mortality [32,41]. There is tension, however, between the policy usefulness of a standard estimate and the representativeness of context specific estimates, each with their own uncertainty range. In particular, the tension between efficiency and distributive equity needs to be carefully considered when assessing the policy usefulness of varied VSL estimates, because, although the willingness-to-pay for specific contexts may vary by the populations affected, it is important to note that income constraints of specific subgroups should not be the determinant of varied VSL recommendations for use in public policy decision-making.

It is unclear whether there is a preference for preventing deaths related to cancer due to the dread and fear associated with cancer deaths and whether a ‘cancer premium’ should be added to the VSL related to cancer mortality [22,27,41]. Despite the ambiguity, some government environmental bodies have recommended higher VSL values for cancer related deaths [29,41]. In a meta-analysis of SP studies conducted by Lindhjem and colleagues [27], VSL from environment related risks were lower than health related risks and there was mixed evidence on any differences between health and traffic related risks. Other SP studies have suggested that VSL from the road context is an underestimate of willingness-to-pay for risk reduction related to environmental risk [32], and that VSL related to health risks are lower than those related to environment and traffic risks [63]. In contrast, a meta-analysis of SP studies by Masterman and Viscusi [35] found that VSL from health, environment and traffic contexts were statistically indistinguishable from each other. How societies value risk reductions in different risk contexts remains an under-researched area [34]. One key issue is the lack of studies investigating VSL across varied risk contexts with the majority of studies investigating the road safety context [34]. Given the impact of study methods on VSL estimates (discussed below), there is a need for future studies to investigate VSL in different risk contexts (health, transport, workplace and environment) using consistent methods. Until further evidence on the preferences of the Australian population is available, we recommend using a consistent VSL across risk contexts. If consideration is given to context specific VSL differences, then these could be undertaken as supplementary analyses with assumptions clearly documented.

Despite the VSLY being a useful measure for valuing public health interventions, there are several issues in its application. The VSLY assumes that each year of life is equally valued. Although this may seem like a reasonable assumption, using a constant VSLY effectively means that VSL steadily declines with age. Although there is no consensus on how age impacts VSL, there is some evidence that VSL for children is higher than adults [22,34,37]; it may follow an inverted U shape in the working age population [27,29,34,35], increasing until around age 40–50 years and then either declining [16,29,34,37] or staying constant in older age. Given this ambiguity [22,27,29,37,41,42], there is no empirical evidence to support the efficiency of using a varied VSL or a constant VSLY. However, as a society, a constant VSLY may be desirable if societal values reflect the ‘fair innings’ argument—higher priority given to interventions impacting younger rather than older age groups because everyone has the right to a ‘normal’ lifespan [64]. This is consistent with one of the rationales for the use of CUA for the evaluation of health interventions, where it is assumed that one of the key goals of health care is to maximise health (often operationalised as maximising healthy life years). In his working paper for the Australian Government Office of Best Practice Regulation, Abelson [16] recommended that choosing a constant VSLY was more attractive than using a constant VSL. Most countries recommend only using varied VSL and a constant VSLY in sensitivity analyses of CBA [61,62]. Further research based on Australian preferences of how VSL and VSLY changes over the lifespan are required. It is important to note that the merits of a policy may differ depending on whether it is evaluated using the VSL or the VSLY, and therefore the merits of using and comparing CBA using VSL and VSLY across different contexts needs further research, discussion and consensus [64].

Another issue with VSLY is the varied estimates derived using different methods. The Australian Government recommendation uses a life expectancy of 40 years to calculate the VSLY from the VSL [16]. The VSLY will vary depending on the life expectancy assumed. It may be more appropriate to calculate the VSLY from individual VSL studies based on the life expectancy of the surveyed population. Empirical evidence also suggests that VSLY derived from the VSL is higher than from studies that directly elicit willingness-to-pay for increases in life expectancy. However there is a paucity of studies that directly elicit the VSLY [18]. Until further Australian evidence is available, we recommend using the updated VSL to calculate a constant VSLY using methods previously used by the Australian Government [16]. The average age of the surveyed population in the Australian studies that inform the calculated central VSLY value was approximately 44 years [44,45], and therefore calculating the VSLY assuming 40 years life expectancy is appropriate [65]. This corresponds to a central value of A$303,531 and a high value of A$315,732. We recommend a low value of A$88,136 based on the only review that specifically investigated the VSLY.

We note that using the VSLY has been recommended as one method for assigning a monetary value to a QALY [16,66]. There is a growing and diverse literature that uses SP techniques to directly elicit the willingness-to-pay for a QALY. A review of this literature found that the value of a QALY elicited from SP studies tended to be lower than when calculated from the VSLY. In addition, the willingness-to-pay values were generally higher if the QALY improvement was based on length of life improvements, compared to quality of life improvements alone. These studies also demonstrated scale bias, similar to SP VSL studies [66]. Other studies have found that the willingness-to-pay for a QALY may not be proportional for illnesses of varied severity and duration, which is inconsistent with using a constant threshold monetary value for all QALYs [67]. It is not clear whether the characteristics of the QALY gain are considered in Australian decision-making, however we do know that decision criteria are wider than simply using a consistent monetary value for a QALY [4]. Although the value or the range of values assigned to QALY gains are not explicit in Australian health policy decision-making, an analysis of prior decisions of the Australian Pharmaceutical Benefits Advisory Committee in 1996 [68] found that the ‘shadow price’ is likely to be between A$68,000 and A$123,000 in 2017 values. For health policy unrelated to pharmaceuticals, the value assigned to additional QALYs when making allocative decisions is unknown. Consistency in decision-making related to health and safety policies requires further research to ascertain the reasons behind the differences in studies eliciting willingness-to-pay for VSL, VSLY and QALYs and to establish whether true differences in preferences exist.

One of the key concerns with using VSL in policy decision-making relates to the methodological issues that threaten the validity of the VSL estimate. Several authors have noted that one of the main sources of variability in VSL relates to estimation methods [31,32,33]. Several studies have reported VSL estimates for their full sample of included studies and for a sample restricted to higher quality studies (although the definition of ‘quality’ varied between papers). The restricted sample resulted in a smaller range and lower VSL estimates [27,34,40]. Although there is no consensus, our review supports other studies that have found that RP studies produce higher VSL estimates than SP studies [22,24,42]. The main methodological issues with RP hedonic wage studies relate to the use of a limited context and population to derive VSL estimates for the general population, the quality of the mortality risk data (for countries outside the US) and the impact of the regression modelling choices made by the analyst [23,30,31,38]. SP studies using contingent valuation surveys have been criticised for issues related to the hypothetical nature of the surveys, their low response rates and the lack of sensitivity of the participant responses to the risk change variable (scale bias) [27,30,34]. Some studies have found that willingness-to-pay is not sensitive to the risk reduction, suggesting that participants have difficulty understanding the small risk changes in the survey [27,34]. Further research is required to improve the understanding of small risks in these surveys, together with more standardised reporting criteria in order to assess whether scale bias is present in contingent valuation studies [22,27,37]. Stated choice experiments are more advanced SP methods that do not rely on participants making trade-offs between money and small mortality risks and better reflect the choices that individuals make in specific contexts [44,45]. However, given that these studies are highly context specific, questions arise as to the validity of transferring estimates from one risk context to another. The base case VSL value recommended from this review is the weighted mean VSL from the two Australian studies [44,45] that used the stated choice technique.

Using a review of the literature to ascertain the VSL for decision-making may be problematic given evidence of publication bias in the reporting of VSL values. Studies found that publication bias favoured higher estimates and correction resulted in lower median VSL values [23,33,35,38,40]. Given these issues (along with the importance of estimates that reflect Australian preferences), we suggest that for Australian decision-making the VSL values from the international literature should be used in sensitivity analyses only.

The key limitation of this study relates to the type of studies included in the systematic review. Earlier we have argued for the importance of Australian data, however, limiting the review to Australian studies was too narrow due to only two studies being published since 2007. Although widening the search to all international primary studies would have provided richer data on the VSL and VSLY, the large number of primary studies internationally made this infeasible. Therefore, we compromised and limited the included studies to primary studies conducted in the Australian context and international review studies.

## 5. Conclusions/Policy Recommendations

This review highlights the need for high quality Australian studies estimating the VSL and the VSLY in different risk contexts. These studies should be updated periodically to reflect any changes in public preferences. Until further data are available, and based on the current literature, we recommend that consideration is given to increasing the base case VSL value used in public policy decision-making in Australia from A$4.3 million to A$7.0 million. This increase is supported by evidence that after controlling for increased income, VSL increases over time [33]. The recommended VSL is lower than that recommended by Viscusi [15], who estimated the appropriate VSL for Australia based on USA RP VSL estimates as approximately A$11.9 million in 2017 values (2015USD 7.9 million). However, according to Abelson [16], the unanswered question remains whether we can afford to use VSL estimates based on public preferences in public policy decisions. The aggregate budget impact of using the VSL and VSLY for health and safety policies is important information for Australian government decision-making, however this remains unknown [16].

In addition to primary VSL, VSLY and willingness-to-pay for QALY estimates for the Australian population across the lifespan, further research should focus on improving methods for eliciting these estimates, assessing how they differ and determining the most appropriate methods to aggregate the estimates for policy decision-making. Synthesis of VSL estimates will also be improved by the development of standard reporting criteria for primary VSL studies and VSL reviews [22].

## Figures and Tables

**Table 3:** VSL values from primary Australian studies (A$2017).

Author	Sample Size	Number of VSL Estimates	Minimum VSL Estimate	Maximum VSL Estimate	Mean/Median VSL Estimate	VSLY Estimate ^a^		
						3% Discount Rate	7% Discount Rate	10% Discount Rate
Hensher et al., 2009 [44]	213	2	7,524,566	7,610,102	7,567,334	327,381	567,619	773,831
Hensher et al., 2011 [45]	99	3	5,071,123	6,394,432	5,829,987	252,219	437,302	596,171
Weighted mean VSL from primary studies ^b^					7,016,060	303,531	526,269	717,458

^a^ The VSLY is calculated using the mean/median estimate; ^b^ The mean weighted by the sample size of each of the studies; A$: Australian dollars; VSL: value of a statistical life; VSLY: value of a statistical life year.

## Data Availability

All data is contained within the article or Supplementary Material.

## Supplementary Materials

The following are available online https://www.mdpi.com/article/10.3390/ijerph18116168/s1, S1: Definition of terms, S2: Systematic literature review search strategy, S3: Equations used in the calculation of the value of a statistical life and the value of a statistical life year, S4: Additional details of VSL estimates from review papers and calculations using various income elasticities; S5: Studies excluded after full text review, S6: Quality assessment of included studies.

## References

[1] Department of Prime Minister and Cabinet, Australian Goverment (2014). The Australian Government Guide to Regulation.

[2] Office of Health Economics (2020). Measuring and Valuing Outcomes.

[3] Organisation for Economic Cooperation and Development (OECD) (2012). Mortality Risk Valuation in Environment, Health and Transport Policies.

[4] Department of Health (2016). Guidelines for Preparing a Submission to the Pharmaceutical Benefits Advisory Committee.

[5] Department of Health, Department of Health (2017). Technical Guidelines for Preparing Assessment Reports for the Medical Services Advisory Committee—Service Type: Investigative (Version 3.0).

[6] Nyborg K. (2014). Project evaluation with democratic decision-making: What does cost–benefit analysis really measure?. Ecol. Econ..

[7] Dobes L., Department of Finance and Deregulation (2007). A Century of Australian Cost-Benefit Analysis.

[8] Swinburn B.A., Sacks G., Hall K.D., McPherson K., Finegood D.T., Moodie M.L., Gortmaker S.L. (2011). The global obesity pandemic: Shaped by global drivers and local environments. Lancet.

[9] Gortmaker S.L., Swinburn B.A., Levy D., Carter R., Mabry P.L., Finegood D.T., Huang T., Marsh T., Moodie M.L. (2011). Changing the future of obesity: Science, policy, and action. Lancet.

[10] World Health Organization (2014). Health in All Policies (HiAP) Framework for Country Action.

[11] Wijnen W., Wesemann P., de Blaeij A. (2009). Valuation of road safety effects in cost-benefit analysis. Eval. Program Plan..

[12] Robinson L.A. (2017). Estimating the values of mortality risk reductions in low- and middle-income countries. J. Benefit Cost Anal..

[13] Majumder A., Madheswaran S. (2017). Meta-analysis of value of statistical life estimates. IIM Kozhikode Soc. Manag. Rev..

[14] Simon N.B., Dockins C., Maguire K.B., Newbold S.C., Krupnick A., Taylor L.O. (2019). Policy Brief—What’s in a Name? A Search for Alternatives to “VSL”. Rev. Environ. Econ. Policy.

[15] Viscusi W.K. (2018). Pricing lives: International guideposts for safety. Econ. Rec..

[16] Abelson P. (2008). Establishing a Monetary Value for Lives Saved: Issues and Controversies.

[17] Abelson P. (2003). The value of life and health for pubic policy. Econ. Rec..

[18] Hein L., Roberts P., Gonzalez L. (2016). Valuing a statistical life year in relation to clean air. J. Environ. Assess. Policy Manag..

[19] Desaigues B., Ami D., Bartczak A., Braun-Kohlová M., Chilton S., Czajkowski M., Farreras V., Hunt A., Hutchison M., Jeanrenaud C. (2011). Economic valuation of air pollution mortality: A 9-country contingent valuation survey of value of a life year (VOLY). Ecol. Indic..

[20] Access Economics (2007). The Health of Nations: The Value of a Statistical Life.

[21] Department of the Prime Minister and Cabinet, Office of Best Practice Regulation (2014). Best Practice Regulation Guidance Note: Value of Statistical Life.

[22] Robinson L.A., Hammitt J.K., O’Keeffe L. (2019). Valuing mortality risk reductions in global benefit-cost analysis. J. Benefit Cost Anal..

[23] Viscusi W.K. (2015). The role of publication selection bias in estimates of the value of a statistical life. Am. J. Health Econ..

[24] Alberini A. (2019). Revealed versus Stated Preferences: What Have We Learned About Valuation and Behavior?. Rev. Environ. Econ. Policy.

[25] Whitty J.A., Lancsar E., Rixon K., Golenko X., Ratcliffe J. (2014). A systematic review of stated preference studies reporting public preferences for healthcare priority setting. Patient Patient Cent. Outcomes Res..

[26] Bridges J.F.P., Hauber A.B., Marshall D., Lloyd A., Prosser L.A., Regier D.A., Johnson F.R., Mauskopf J. (2011). Conjoint analysis applications in health—A Checklist: A report of the ISPOR Good Research Practices for Conjoint Analysis task force. Value Health.

[27] Lindhjem H., Navrud S., Braathen N.A., Biausque V. (2011). Valuing mortality risk reductions from environmental, transport, and health policies: A global meta-analysis of stated preference studies. Risk Anal. Int. J..

[28] Baethge C., Goldbeck-Wood S., Mertens S. (2019). SANRA—A scale for the quality assessment of narrative review articles. Res. Integr. Peer Rev..

[29] Andersson H., Treich N. (2008). The Value of a Statistical Life.

[30] Bahamonde-Birke F.J., Kunert U., Link H. (2015). The value of a statistical life in a road safety context—A review of the current literature. Transp. Rev..

[31] Bellavance F., Dionne G., Lebeau M. (2009). The value of a statistical life: A meta-analysis with a mixed effects regression model. J. Health Econ..

[32] Dekker T., Brouwer R., Hofkes M., Moeltner K. (2011). The effect of risk context on the value of a statistical life: A Bayesian meta-model. Environ. Resour. Econ..

[33] Doucouliagos C., Stanley T.D., Giles M. (2012). Are estimates of the value of a statistical life exaggerated?. J. Health Econ..

[34] Hultkrantz L., Svensson M. (2012). The value of a statistical life in Sweden: A review of the empirical literature. Health Policy.

[35] Masterman C.J., Kip Viscusi W. (2018). The income elasticity of global values of a statistical life: Stated preference evidence. J. Benefit Cost Anal..

[36] Milligan C., Kopp A., Dahdah S., Montufar J. (2014). Value of a statistical life in road safety: A benefit-transfer function with risk-analysis guidance based on developing country data. Accid. Anal. Prev..

[37] Robinson L.A., Hammitt J.K. (2016). Valuing reductions in fatal illness risks: Implications of recent research. Health Econ..

[38] Viscusi W.K., Masterman C. (2017). Anchoring biases in international estimates of the value of a statistical life. J. Risk Uncertain..

[39] Viscusi W.K., Aldy J.E. (2003). The value of a statistical life: A critical review of market estimates throughout the world. J. Risk Uncertain..

[40] Wheeler W., Dockins C. (2013). Meta-Analysis and Publication Bias in the Hedonic Wage Literature.

[41] U.S. Environmental Protection Agency, National Center for Environmental Economics (2010). Valuing Mortality Risk Reductions for Environmental Policy: A White Paper.

[42] Yaduma N., Kortelainen M., Wossink A. (2013). Estimating mortality and economic costs of particulate air pollution in developing countries: The case of Nigeria. Environ. Resour. Econ..

[43] Miller T.R. (2000). Variations between countries in values of statistical life. J. Transp. Econ. Policy.

[44] Hensher D.A., Rose J.M., Ortuzar J.d.D., Rizzi L.I. (2009). Estimating the willingness to pay and value of risk reduction for car occupants in the road environment. Transp. Res. Part A Policy Pract..

[45] Hensher D.A., Rose J.M., De Dios Ortúzar J., Rizzi L.I. (2011). Estimating the value of risk reduction for pedestrians in the road environment: An exploratory analysis. J. Choice Model..

[46] Kniesner T., Leeth J.D. (1991). Compensating wage differentials for fatal injury risk in Australia, Japan, and the United States. J. Risk Uncertain..

[47] Miller P., Mulvey C., Norris K. (1997). Compensating differentials for risk of death in Australia. Econ. Rec..

[48] The World Bank (2019). GNI per Capita, Atlas Method (Current US$). https://data.worldbank.org/indicator/NY.GNP.PCAP.CD?end=2017&name_desc=false&start=2005.

[49] Organisation for Economic Cooperation and Development (OECD) (2014). PPPs and Exchange Rates. https://www.oecd-ilibrary.org/content/data/data-00004-en.

[50] Australian Institute of Health and Welfare (2017). Health Expenditure Australia 2015–16.

[51] Dobes L., Leung J., Argyrous G. (2016). Social Cost-Benefit Analysis in Australia and New Zealand: The State of Current Practice and What Needs to Be Done.

[52] Department of Prime Minister and Cabinet, Australian Goverment (2016). Cost-Benefit—Guidance Note.

[53] Organisation for Economic Cooperation and Development (OECD) (2019). Gross National Income. https://data.oecd.org/natincome/gross-national-income.htm.

[54] Viscusi W.K., Masterman C.J. (2017). Income elasticities and global values of a statistical life. J. Benefit Cost Anal..

[55] Doucouliagos H., Stanley T.D., Viscusi W.K. (2014). Publication selection and the income elasticity of the value of a statistical life. J. Health Econ..

[56] Australian Institute of Health and Welfare (2018). Health Expenditure Australia 2016–17.

[57] Clough P., Guria J., Bealing M. (2015). Approaches To Valuing Injury and Mortality Risk in Transport Assessments.

[58] Kniesner T.J., Leeth J.D. (2010). Hedonic wage equilibrium: Theory, evidence and policy. Found. Trends Microecon..

[59] Cowgill M. (2019). Which Countries Are Most Similar to Australia? Some Answers Might Surprise You.

[60] Ministry of Transport (2019). Social Cost of Road Crashes And Injuries 2018 Update.

[61] Chestnut L.G., De Civita P. (2009). Economic Valuation of Mortality Risk Reduction: Review and Recommendations for Policy and Regulatory Analysis.

[62] Organisation for Economic Cooperation and Development (OECD) (2011). Valuing Mortality Risk Reduction in Regulatory Ananlysis of Environmental, Health and Transport Policies: Poliy Implications.

[63] Braathen N., Lindhjem H., Navrud S. Valuing Lives Saved from Environmental, Transport and Health Policies: A Meta-Analysis of Stated Preference Studies. Proceedings of the Managing the Social Impacts of Change from a Risk Perspective.

[64] Hammitt J.K. (2007). Valuing changes in mortality risk: Lives saved versus life years saved. Rev. Environ. Econ. Policy.

[65] Australian Bureau of Statistics 3302.0.55.001—Life Tables, States, Territories and Australia, 2016–2018. https://www.abs.gov.au/ausstats/abs@.nsf/mf/3302.0.55.001.

[66] Ryen L., Svensson M. (2015). The Willingness to Pay for a Quality Adjusted Life Year: A Review of the Empirical Literature. Health Econ..

[67] Hammitt J.K., Haninger K. (2017). Valuing nonfatal health risk as a function of illness severity and duration: Benefit transfer using QALYs. J. Environ. Econ. Manag..

[68] George B., Harris A., Mitchell A. (2001). Cost-effectiveness analysis and the consistency of decision making. Pharmacoeconomics.

