# Supplementary material for: Systematic Review to Update ‘Value of a Statistical Life’ Estimates for Australia
Source: Int J Environ Res Public Health. 2021 Jun 7;18(11):6168. doi: 10.3390/ijerph18116168 (PMC8201370; doi:10.3390/ijerph18116168)
Supplement: Supplementary file 1 [file ijerph-18-06168-s001.zip › ijerph-1250389-supplementary.pdf]

S1: Definition of terms

Table S1: Definition of terms

Benefit/value transfer function	A function developed to make appropriate adjustments to the value of a statistical life value (or other economic value) from one study in order to make it applicable to the new country or context. This is often done by undertaking a meta-regression.[1]
Contingent valuation	Survey based technique to value non-market goods by constructing hypothetical markets. The surveys directly elicit participants' willingness-to-pay for these goods.[2]
Cost-benefit analysis	Economic evaluation technique used to assess the costs and benefits of various courses of action, where the benefits and the costs are monetised.[3]
Cost-effectiveness analysis	Economic evaluation technique used to assess the costs and benefits of various courses of action, where the benefits are estimated in physical units, e.g. cost per case of cancer prevented.[3]
Cost-utility analysis	Economic evaluation technique used to assess the costs and benefits of various courses of action, where the benefits are estimated using a generic measure of health that incorporates the quality and quantity of life gained, e.g. cost per quality adjusted life year gained.[4]
Disability adjusted life years	A generic measure of health burden that incorporates the years of life lost due to premature mortality and years lost due to disability.[4]
Hedonic wage studies	Studies that use the labour market to estimate the compensating wage differential for a worker accepting a small increase in work related fatality risk.[5]
Income elasticity of willingness to pay for mortality reduction	Measure of the sensitivity of the willingness to pay for a good (mortality reduction) to income. The income elasticity of willingness to pay for mortality reduction is often estimated using meta-regression to estimate how the value of a statistical life changes as income changes.[6]
Publication bias	Bias introduced when relying on published academic research where the result of the research influences whether the research is published. Several techniques can be used to adjust for publication bias and provide bias corrected estimates.[7]

Purchasing power parity	Estimates the rate of currency conversion that equalises the purchasing power of different currencies by eliminating the differences in price levels between countries.[2]
Quality adjusted life year	A generic measure of health where the length of time affected by a condition is adjusted for quality of life during that period.[4]
Revealed preference studies	Valuation of goods based on individual behaviour in markets where prices reflect differences in mortality risk.[1]
Scale bias/scale effect/scope test in contingent valuation studies	Respondents' willingness-to-pay for risk reduction should be sensitive and near proportional to the size of the risk. Scale bias occurs when participants are not sensitive to the size of the risk reducing the validity of the results. Scope tests assess the presence of scale bias or scale effects.[1, 8]
Stated choice experiment	Survey based technique that elicits valuations of a good based on participants' stated choice between goods with different levels of attributes.[9]
Stated preference studies	Valuation of non-market goods based on surveys of people's stated preferences and their willingness to pay for hypothetical scenarios.[1, 3]
Value of a statistical life	An estimate of individual willingness to trade wealth for fatality risk reductions.[10] It is a key parameter for the evaluation of public policies related to health and safety.
Value of a statistical life year	The monetised value of one additional year of life expectancy.[11] This can be calculated using the value of a statistical life or using stated preference studies.

References

1. Organisation for Economic Cooperation and Development (OECD). Mortality risk valuation in environment, health and transport policies: Organisation for Economic Cooperation and Development (OECD); 2012. 1-139 p.
2. Organisation for Economic Cooperation and Development (OECD). Glossary of Statistical Terms: Organisation for Economic Cooperation and Development (OECD); 2020 [cited 2020]. Available from: https://stats.oecd.org/glossary/index.htm.
3. Commonwealth of Australia. Introduction to cost-benefit analysis and alternative evaluation methodologies: Dept. of Finance and Administration; 2006.
4. Drummond MF, Sculpher MJ, Claxton K, Stoddart GL, Torrance GW. Methods for the Economic Evaluation of Health Care Programmes. Oxford, UNITED KINGDOM: Oxford University Press; 2015.

5. Majumder A, Madheswaran S. Meta-analysis of value of statistical life estimates. IIM Kozhikode Society & Management Review. 2017;6(1):110-20. doi: http://dx.doi.org/10.1177/2277975216678546. PubMed PMID: 1929785525.
6. Doucouliagos H, Stanley TD, Viscusi WK. Publication selection and the income elasticity of the value of a statistical life. Journal of Health Economics. 2014;33:67-75. doi: 10.1016/j.jhealeco.2013.10.010. PubMed PMID: 93593111.
7. Dubben H-H, Beck-Bornholdt H-P. Systematic review of publication bias in studies on publication bias. BMJ. 2005;331(7514):433-4. doi: 10.1136/bmj.38478.497164.F7.
8. Bahamonde-Birke FJ, Kunert U, Link H. The value of a statistical life in a road safety context - A review of the current literature. Transport Reviews. 2015;35(4):488. PubMed PMID: 1688627986.
9. Hensher DA, Rose JM, Ortuzar JdD, Rizzi LI. Estimating the willingness to pay and value of risk reduction for car occupants in the road environment. Transportation Research: Part A: Policy and Practice. 2009;43(7):692-707. doi: http://www.sciencedirect.com/science/journal/09658564. PubMed PMID: 1059819.
10. Robinson LA. Estimating the values of mortality risk reductions in low- and middle-income countries. Journal of Benefit-Cost Analysis. 2017;8(2):205-14. Epub 08/10. doi: 10.1017/bca.2017.14.
11. Hein L, Roberts P, Gonzalez L. Valuing a statistical life year in relation to clean air. Journal of Environmental Assessment Policy & Management. 2016;18(4):-1. doi: 10.1142/S1464333216500253. PubMed PMID: 120530328.

S2: Systematic literature review search strategy

Table S2: Systematic literature review search strategy

Database	**JSTOR**<br>Results for each search were exported.
Date of search	10 March 2017, updated 23 January 2019
S1	(ti:"value of life" OR tb:"value of life" OR ti:"value of life" OR tb:"value of life") AND la:(eng OR en)
S2	(((ti:"value statistical"~5 OR tb:"value statistical"~5) AND (ti:"statistical life"~5 OR tb:"statistical life"~5) AND (ti:life OR tb:life)) OR (ab:"value statistical"~5 AND ab:"statistical life"~5)) AND la:(eng OR en)
S3	(ti:VSL OR tb:VSL OR ab:VSL) AND la:(eng OR en)
S4	(((ti:"value risk"~5 OR tb:"value risk"~5) AND (ti:"risk reduction"~5 OR tb:"risk reduction"~5) AND (ti:reduction OR tb:reduction)) OR ti:VRR OR tb:VRR OR ab:VRR) AND la:(eng OR en)
S5	(((ti:"willingness to pay" OR tb:"willingness to pay") AND (ti:health OR tb:health)) OR ab:"willingness to pay") AND ab:health AND la:(eng OR en)
S6	(((ti:"willingness to pay" OR tb:"willingness to pay") AND (ti:life OR tb:life)) OR ab:"willingness to pay") AND ab:life AND la:(eng OR en)
S7	(((ti:"willingness to pay" OR tb:"willingness to pay") AND (ti:death OR tb:death)) OR ab:"willingness to pay") AND ab:death AND la:(eng OR en)
S8	(((ti:"willingness to pay" OR tb:"willingness to pay") AND (ti:fatal* OR tb:fatal*)) OR ab:"willingness to pay") AND ab:fatal* AND la:(eng OR en)
S9	(((ti:"willingness to pay" OR tb:"willingness to pay") AND (ti:injury OR tb:injury)) OR ab:"willingness to pay") AND ab:injury AND la:(eng OR en)
Limiters	Publication date: 1 January 2007 to January 22 2019<br>Language: English

Database	**Science Direct**

	S1 shows the search strategy for search undertaken in March 2017. Individual searches were run on January 24th as the platform no longer supported wildcards and proximity terms.
Date of search	13 March 2017, updated 24 January 2019
S1	(pub-date > 2006 and TITLE-ABSTR-KEY("value of life")) OR ((pub-date > 2006 and TITLE-ABSTR-KEY(value W/5 "statistical life") or TITLE-ABSTR-KEY(value W/5 "statistical lives")) OR (pub-date > 2006 and pub-date > 2006 and TITLE-ABSTR-KEY(VSL) and not TITLE-ABSTR-KEY(VSL#3))) OR (pub-date > 2006 and pub-date > 2006 and TITLE-ABSTR-KEY(value W/5 "risk reduction") or TITLE-ABSTR-KEY(VRR)) OR ((pub-date > 2006 and TITLE-ABSTR-KEY("willingness to pay") or TITLE-ABSTR-KEY(WTP)) AND (pub-date > 2006 and TITLE-ABSTR-KEY(life or death or fatal* or injury or health*))).
Limiters	Publication date: 2007 or later

Database	**ProQuest**
Date of search	14 March 2017, updated 24 January 2019
S1	((ti((value NEAR/5 "risk reduction") OR VRR) OR ab((value NEAR/5 "risk reduction") OR VRR) OR pub((value NEAR/5 "risk reduction") OR VRR)) AND la.exact("English")) OR (((ti((value NEAR/5 ("statistical life" OR "statistical lives")) OR VSL) OR ab((value NEAR/5 ("statistical life" OR "statistical lives")) OR VSL) OR pub((value NEAR/5 ("statistical life" OR "statistical lives")) OR VSL)) AND la.exact("English")) NOT ((ti(vsl3 OR vsl#3) OR ab(vsl3 OR vsl#3) OR pub(vsl3 OR vsl#3)) AND la.exact("English"))) OR ((ti(("Willingness to pay" OR WTP) NEAR/5 (death OR life OR fata* OR injury OR health*)) OR ab(("Willingness to pay" OR WTP) NEAR/5 (death OR life OR fata* OR injury OR health*)) OR pub(("Willingness to pay" OR WTP) NEAR/5 (death OR life OR fata* OR injury OR health*))) AND la.exact("English")) OR ((ti("value of life") OR ab("value of life") OR pub("value of life")) AND la.exact("English"))
Limiters	Publication date: Publication date: 1 January 2007 to January 22 2019<br>Language: English

Database	**SCOPUS**

Date of search	13 March 2017, updated 24 January 2019
S1	LANGUAGE (english) AND TITLE-ABS-KEY ("value of life") AND PUBYEAR > 2016
S2	LANGUAGE (english) AND TITLE-ABS-KEY((value W/5 ("statistical life" OR "statistical lives"))) OR TITLE-ABS-KEY(vsl AND NOT (vsl#3 OR vsl3)) AND PUBYEAR > 2006
S3	LANGUAGE (english) AND TITLE-ABS-KEY ((value W/5 "risk reduction") OR vrr) AND PUBYEAR > 2006
S4	LANGUAGE (english) AND TITLE-ABS-KEY((willingness W/5 pay OR wtp) W/5 (life OR death OR fata* OR injury OR health*)) AND PUBYEAR > 2006
Limiters	Publication date: 2007 or later<br>Language: English

Database	**Informit**
Date of search	13 March 2017, updated 24 January 2019
S1	((TI:VVR OR AB:VRR) OR ((TI:"value of life") OR (AB:"value of life"))) OR (((TI:health OR AB:health) OR (TI:injury OR AB:injury) OR (TI:fatal* OR AB:fatal*) OR (TI:death OR AB:death) OR (TI:life OR AB:life)) AND ((TI:"willingness to pay") OR (AB:"willingness to pay"))) OR ((TI:VSL OR AB:VSL) OR ((TI:value TI:statistical TI:life) OR (AB:value AB:statistical AB:life)))
Limiters	Publication date: 2007 -2019

Database	**Embase**
Date of search	14 March 2017, updated 24 January 2019
	(vrr:ab,ti AND english:la OR (((value NEAR/5 'risk reduction'):ab,ti) AND english:la) OR (((value NEAR/5 'statistical life'):ab,ti) AND english:la) OR ('vsl':ab,ti NOT vsl3:ab,ti AND english:la) OR ('willingness to pay':ab,ti AND english:la AND (life:ab,ti AND english:la OR (death:ab,ti AND english:la) OR (injury:ab,ti AND english:la) OR (health:ab,ti AND english:la) OR (fata*:ab,ti AND english:la))) OR

	(((value NEAR/5 life):ab,ti) AND english:la)) AND [embase]/lim AND [2007-2019]/py
Limiters	Publication date: 2007 -2019<br>Language: English

Database	**EBSCOhost interface**<br>The search was run individually for the following databases: Academic Search Complete, Business Source Complete, CINHAL Complete, EconLit, Environment Complete, Global Health, GreenFILE, Health Business Elite, Health Policy Reference Center, Health Source: Nursing/Academic Edition, MEDLINE Complete, Political Science Complete, and PsycINFO. The search terms and limiters varied slightly across the databases
Date of search	10 March 2017, updated 22 January 2019
Search modes	Boolean/Phrase in title, abstract and keywords
S1	TI "value of life" OR AB "value of life" OR KW "value of life"
S2	TI (value N5 ("statistical life" or "statistical lives")) OR AB (value N5 ("statistical life" or "statistical lives")) OR KW (value N5 ("statistical life" or "statistical lives"))
S3	TI (VSL NOT (VSL#3)) OR AB (VSL NOT (VSL#3)) OR KW (VSL NOT (VSL#3))
S4	S2 OR S3
S5	TI value N5 "risk reduction" OR AB value N5 "risk reduction" OR KW value N5 "risk reduction"
S6	TI "willingness to pay" OR AB "willingness to pay" OR KW "willingness to pay"
S7	TI life OR AB life OR KW life
S8	TI death OR AB death OR KW death
S9	TI fata* OR AB fata* OR KW fata*
S10	TI injury OR AB injury OR KW injury
S11	TI health* OR AB health* OR KW health*

S12	S7 OR S8 OR S9 OR S10 OR S11
S13	TI VRR OR AB VRR OR KW VRR
S14	S5 OR S13
S15	TI wtp OR AB wtp OR KW wtp
S16	(S6 OR S15)
S17	S16 N5 S12
S18	S1 OR S4 OR S14 OR S17
Limiters	Publication date: 1 January 2007 to January 22 2019<br>Language: English

S3: Equations used in the calculation of the value of a statistical life and the value of a statistical life year

Equation 1: Value of a statistical life year (VSLY) calculation

VSLY = VSL/A

Where A is

A = $\frac{1-(1+r)^{-n}}{r}$

Where n is the discount period (we have assumed 40 years), and r is the discount rate.

Equation 2: Australian value of a statistical life (VSL) calculation

$$VSL_{AUS} = VSL_{INT_x} \times \left(\frac{GNI_{AUS_x}}{GNI_{INT_x}}\right)^e \times \left(\frac{PPP_{AUS_x}}{PPP_{INT_x}}\right) \times \left(\frac{GDP_{2017}}{GDP_x}\right)$$

Where VSL_{AUS} is the VSL for Australia in year 2017; VSL_{INT} is the estimate reported in an international currency in year x; the ratio of GNI_{AUS} to GNI_{INT} is the gross national income per capita for Australia and the international estimate respectively for year x; e is the elasticity of willingness to pay for reductions in mortality relative to income; PPP_{AUS_x} and PPP_{INT_x} are purchasing power parities for Australia and the international estimate respectively in year x which takes into account the differences in currency; GDP_{2017} and GDP_x are the gross domestic product price deflator index values for 2017 and the estimate year x respectively.

When the review studies report VSL estimates from several countries, the GNI and PPP for each of the estimates are used, however if this information is missing, the currency that the study is reported in is used.

S4: Additional details of VSL estimates from review papers and calculations using various income elasticities

Table S4: Additional details of VSL estimates from review papers and calculations using various income elasticities

Study	Countries included	Country for median VSL estimate	Median VSL estimate using various income elasticities [a, b]		
			0.3	0.7	1.2
**Stated Preference Studies**					
Bahamonde-Birke et al 2015 (1)	Australia, Chile, Czech Republic, Norway, Spain, Sweden	Australia[c]	Not applicable		
Dekker et al 2011 (2)	Italy, Sweden	Sweden	2,264,665	1,925,352	1,571,784
Hein et al 2016 (3)	Czech Republic, Denmark, France, Germany, Hungary, Poland, Spain, Switzerland, UK,	Denmark, France, Germany, Spain, Switzerland, UK,	Not applicable		
Hultkrantz & Svensson 2012 (4)	Sweden	Sweden	9,308,894	8,145,427	6,893,401
Lindhjem et al 2011 (5)	Czech Republic, Italy, Switzerland, United Kingdom	Switzerland	6,549,413	5,322,046	4,106,048
Robinson & Hammitt 2016 (6)	USA	USA	13,396,119	14,515,149	16,046,290
Robinson et al 2019 (7)	Bangladesh, Brazil, China, India, Malaysia, Mongolia, Poland, Sudan, Thailand, Turkey,	Thailand	1,516,738	3,832,248	12,207,665
Milligan et al 2014 (8)	Mongolia, South Korea, USA	USA	16,818,115	15,810,506	14,635,458

**Revealed Preference Studies**					
Hultkrantz & Svensson 2012 (4)	Sweden	Sweden	6,229,709	5,829,563	5,365,328
Robinson & Hammitt 2016 (6)	USA	USA	13,553,721	14,685,916	16,235,070
Robinson et al 2019 (7)	Chile, China, India, Pakistan, Poland, Tunisia, Turkey	Tunisia, Turkey	5,074,990	9,867,187	25,364,840
Viscusi & Masterman 2017 (9) [d]	Chile, Germany, Japan, Pakistan, Poland, Taiwan	Poland, Germany	3,789,995	3,642,172	3,467,563
	USA	USA	17,178,496	17,623,076	18,195,014
Wheeler & Dockins 2013 (10) [e]	USA	USA	11,974,122	9,664,525	7,393,525
**Stated and Revealed Preference Studies**					
Yaduma et al 2013 (11)	China, India, Mexico, Thailand	India, Thailand	673,198	1,524,296	4,477,556

[a] International studies have been translated to Australian values by adjusting for income using The World Bank Gross National Index (GNI) values (12), converted to A$ using OECD purchasing power parities (PPP) (13) and inflated to 2017 values using the gross domestic product price deflator index values.(14)

[b] The impact of the income elasticity varies depending on the income of the country the median estimate is from. If the estimate used is from a country with a higher income compared to Australia, an income elasticity below 1 results in a higher estimate and an elasticity over 1 results in a lower estimate. The opposite occurs if the estimates used is from a country with a lower income to Australia.

[c] The median estimate is from an Australian study and therefore various income elasticities are not relevant.

[d] The first row represents values from international studies. The second row presents values used to estimate the VSL using Census of Fatal Occupational Injuries (CFOI) data. Primary data used to calculate the VSL was not reported. The median value for this CFOI dataset is reported here.

[e] The VSL is the publication bias corrected estimate.

A$: Australian dollars; CFOI: Census of Fatal Occupational Injuries; GNI: Gross National Income; OECD: Organisation for Economic Co-operation and Development; RP: revealed preference; VSL: value of a statistical life

References

1. Bahamonde-Birke FJ, Kunert U, Link H. The value of a statistical life in a road safety context - A review of the current literature. Transport Reviews. 2015;35(4):488.
2. Dekker T, Brouwer R, Hofkes M, Moeltner K. The effect of risk context on the value of a statistical life: A Bayesian meta-model. Environmental and Resource Economics. 2011;49(4):597-624.
3. Hein L, Roberts P, Gonzalez L. Valuing a statistical life year in relation to clean air. Journal of Environmental Assessment Policy & Management. 2016;18(4):-1.
4. Hultkrantz L, Svensson M. The value of a statistical life in Sweden: A review of the empirical literature. Health Policy. 2012;108(2/3):302-10.
5. Lindhjem H, Navrud S, Braathen NA, Biausque V. Valuing mortality risk reductions from environmental, transport, and health policies: A global meta-analysis of stated preference studies. Risk Analysis: An International Journal. 2011;31(9):1381-407.
6. Robinson LA, Hammitt JK. Valuing reductions in fatal illness risks: Implications of recent research. Health Economics. 2016;25(8):1039-52.
7. Robinson LA, Hammitt JK, O'Keeffe L. Valuing mortality risk reductions in global benefit-cost analysis. Journal of Benefit-Cost Analysis. 2019(S1):15-50.
8. Milligan C, Kopp A, Dahdah S, Montufar J. Value of a statistical life in road safety: A benefit-transfer function with risk-analysis guidance based on developing country data. Accident Analysis & Prevention. 2014;71:236-47.
9. Viscusi WK, Masterman C. Anchoring biases in international estimates of the value of a statistical life. Journal of Risk & Uncertainty. 2017;54(2):103-28.
10. Wheeler W, Dockins C. Meta-analysis and publication bias in the hedonic wage literature. St. Louis: Federal Reserve Bank of St Louis; 2013 2013.

11. Yaduma N, Kortelainen M, Wossink A. Estimating mortality and economic costs of particulate air pollution in developing countries: The case of Nigeria. Environmental and Resource Economics. 2013;54(3):361-87.
12. GNI per capita, Atlas method (current US$) [Internet]. 2019 [cited 26 April 2019]. Available from: https://data.worldbank.org/indicator/NY.GNP.PCAP.CD?end=2017&name_desc=false&start=2005.
13. PPPs and exchange rates [Internet]. 2014 [cited 26 April 2019]. Available from: https://www.oecd-ilibrary.org/content/data/data-00004-en.
14. Australian Institute of Health and Welfare. Health expenditure Australia 2015–16. Canberra: Australian Institute of Health and Welfare; 2017.

S5: Studies excluded after full text review

Table S5: Studies excluded after full text review

	**Reference**	**Reason for exclusion**
1	Alberini A. Measuring the economic value of the effects of chemicals on ecological systems and human health. *OECD Environment Working Papers.* 2017(116):1-46.	Not a review of VSL
2	Aldy JE, Smyth SJ. Heterogeneity in the Value of Life. *National Bureau of Economic Research Working Paper Series.* 2014;No. 20206.	Not a review of VSL
3	Aldy JE, Viscusi WK. Symposium: Mortality-Risk Valuation and Age: Age Differences in the Value of Statistical Life: Revealed Preference Evidence. *Review of Environmental Economics and Policy.* 2007;1(2):241-260.	Not a review of VSL
4	Ambrey CL, Fleming CM, Manning M. The social cost of the Black Saturday bushfires. *Australian Journal of Social Issues (John Wiley & Sons, Inc).* 2017;52(4):298-312.	Not a primary study reporting VSL for Australia
5	Batabyal AA, Nijkamp P. *Research Tools in Natural Resource and Environmental Economics*. Hackensack, N.J. and Singapore: World Scientific; 2011.	Not a review of VSL
6	Bauer D, Lakdawalla D, Reif J. Mortality Risk, Insurance, and the Value of Life. In: National Bureau of Economic Research, Inc, NBER Working Papers: 25055; 2018.	Not a review of VSL
7	Becker GS. Health as human capital: synthesis and extensions. *Oxford Economic Papers.* 2007;59(3):379-410.	Not a review of VSL
8	Blomquist GC. Value of Life, Economics of A2 - Wright, James D. In: *International Encyclopedia of the Social & Behavioral Sciences (Second Edition).* Oxford: Elsevier; 2015:14-20.	Not a review of VSL

9	Bockarjova M, Rietveld P, Verhoef ET. Composite Valuation of Immaterial Damage in Flooding: Value of Statistical Life, Value of Statistical Evacuation and Value of Statistical Injury. In: Tinbergen Institute, Tinbergen Institute Discussion Papers: 12-047/3; 2012.	Not a review of VSL
10	Braathen N, Lindhjem H, Navrud S. *Valuing Lives Saved from Environmental, Transport and Health Policies: A Meta-Analysis of Stated Preference Studies.* 2009.	Working paper using data reported in a paper included in this review
11	Brey R, Pinto-Prades JL. Age Effects in Mortality Risk Valuation. *European Journal of Health Economics.* 2017;18(7):921-932.	Not a review of VSL
12	Chang AY, Robinson LA, Hammitt JK, Resch SC. Economics in "Global Health 2035": a sensitivity analysis of the value of a life year estimates. *Journal Of Global Health.* 2017;7(1):010401-010401.	Not a review of VSL
13	Clough P, Guria J, Bealing M. *Approaches to valuing injury and mortality risk in transport assessments.* Wellington, New Zealand: New Zealand Transport Agency; 2015-08 2015. 9780478445152	No data on VSL from different studies
14	Courard-Hauri D, Lauer SA. Taking 'All Men Are Created Equal' Seriously: Toward a Metric for the Intergroup Comparison of Utility Functions through Life Values. *Journal of Benefit-Cost Analysis.* 2012;3(3).	Not a review of VSL
15	Cropper M, Hammitt JK, Robinson LA. Valuing Mortality Risk Reductions: Progress and Challenges. *Annual Review of Resource Economics.* 2011;3(1):313-336.	Not a review of VSL
16	Cropper ML, Sahin S. Valuing mortality and morbidity in the context of disaster risks. In. *Policy Research Working Paper - World Bank*. Washington; USA: World Bank; 2009.	Not a review of VSL
17	Doucouliagos H, Stanley TD, Viscusi WK. Publication selection and the income elasticity of the value of a statistical life. *Journal of Health Economics.* 2014;33:67-75.	Not a review of VSL
18	Fifer S, Greaves S, Rose J, Ellison R. A combined GPS/Stated Choice experiment to estimate values of crash-risk reduction. *Journal of Choice Modelling.* 2011;4(1):44-61.	Not a primary study reporting VSL for Australia

19	Fischer S. What are you worth? *New Scientist.* 2016(3096):28-33.	Not a review of VSL
20	Fistung FD. New Approach for Monetary Valuation of the Statistical Life. *Acta Universitatis Danubius: Oeconomica.* 2017;13(4):250-263.	Not a review of VSL
21	Hultkrantz L, Svensson M. The Economic Value of Preventing Fatalities: Recent evidence on the value of a statistical life in Sweden. In. St. Louis: Federal Reserve Bank of St Louis; 2010.	Working paper of a published paper included in this review
22	International Road Assessment Programme. *The true cost of road crashes: valuing life and the cost of a serious injury.* Basingstoke, United Kingdom: International Road Assessment Programme (iRAP); 2008 2008.	Not a review of VSL
23	Jaafar H, Razi NA, Azzeri A, Isahak M, Dahlui M. A systematic review of financial implications of air pollution on health in Asia. *Environmental Science & Pollution Research.* 2018;25(30):30009-30020.	Not a review of VSL
24	Jones-Lee M, Spackman M. The development of road and rail transport safety valuation in the United Kingdom. *Research in Transportation Economics.* 2013;43(1):23-40.	Not a review of VSL
25	Kluve J, Schaffner S. The Value of Life in Europe - A Meta-Analysis. *Sozialer Fortschritt.* 2008;57(10/11):279-287.	German publication, only abstract in English
26	Kniesner TJ, Leeth JD. Hedonic Wage Equilibrium: Theory, Evidence and Policy. *Foundations and Trends in Microeconomics.* 2010;5(4):229-299.	Not a review of VSL
27	Kniesner TJ, Leeth JD. Value of a Statistical Life A2 - Shogren, Jason F. In: *Encyclopedia of Energy, Natural Resource, and Environmental Economics.* Waltham: Elsevier; 2013:359-367.	Not a review of VSL
28	Kniesner TJ, Viscusi WK, Woock C, Ziliak JP. The Value of a Statistical Life: Evidence from Panel Data. In. St. Louis: Federal Reserve Bank of St Louis; 2010.	Not a review of VSL

29	Krupnick A. Symposium: Mortality-Risk Valuation and Age: Mortality-Risk Valuation and Age: Stated Preference Evidence. *Review of Environmental Economics and Policy.* 2007;1(2):261-282.	Review limited to studies investigating the relationship between age and VSL
30	Majumder A, Madheswaran S. Compensating Wage Differential and Value of Statistical Life: A Meta-analysis. *Indian Journal of Labour Economics*. 2017;60(4):527-548.	Unable to calculate VSL values for individual studies from data reported.
31	Majumder A, Madheswaran S. Meta-analysis of Value of Statistical Life Estimates. *IIM Kozhikode Society & Management Review.* 2017;6(1):110-120.	Unable to calculate VSL values for individual studies from data reported.
32	McDonald R, Chilton S, Jones-Lee M, Metcalf H. Dread and latency impacts on a VSL for cancer risk reductions. *Journal of Risk & Uncertainty.* 2016;52(2):137-161.	Not a review of VSL
33	McGartland A, Revesz R, Axelrad DA, Dockins C, Sutton P, Woodruff TJ. Estimating the health benefits of environmental regulations: Changes needed for complete benefits assessment. *Science.* 2017;357(6350):457-458.	Not a review of VSL
34	Mrozek JR, Taylor LO. What Determines the Value of Life: A Meta-Analysis. In: *Cost-Benefit Analysis and Public Policy.* Blackwell Publishing Ltd.; 2009:161-178.	Originally published in 2002.
35	Naude C, Makwasha T, McGeehan E. Methodologies for updating parameter values for economic evaluation of road transport projects in Australia. Analytical presented at Australasian Transport Research Forum (ATRF), 37th, 2015, Sydney, New South Wales, Australia; 2015-09, 2015; Canberra, ACT, Australia.	Not a primary study reporting VSL for Australia
36	Nelson JP, Kennedy PE. The Use (and Abuse) of Meta-analysis in Environmental and Natural Resource Economics: An Assessment. *Environmental and Resource Economics.* 2009;42(3):345-377.	Not a review of VSL
37	Organisation for Economic Cooperation and Development (OECD). *Mortality risk valuation in environment, health and transport policies.* Vol 9789264130807: Organisation for Economic Cooperation and Development (OECD); 2012.	Report of a published paper included in this review

38	Perovic J, Tsolakis D. Valuing the social costs of crashes: is community's willingness to pay to avoid death or injury being reflected? Analytical presented at Australasian Road Safety Research Policing Education Conference, 2008, Adelaide, South Australia, Australia; 2008-11, 2008; Walkerville, South Australia, Australia.	Not a primary study reporting VSL for Australia
39	Prosser LA, Hammitt JK, Keren R. Measuring Health Preferences for Use in Cost-Utility and Cost-Benefit Analyses of Interventions in Children. *PharmacoEconomics.* 2007;25(9):713-726.	Not a review of VSL
40	Rafiq M, Shah MK, Gul S, Rahman AU. On the value of statistical life and injury: A review. *Eur J Econ Financ Adm Sci.* 2010(25):7-15.	Unable to access the full article
41	Rheinberger CM, Herrera-Araujo D, Hammitt JK. The value of disease prevention vs treatment. *Journal of Health Economics.* 2016;50:247-255.	Not a review of VSL
42	Risbey T, Cregan M, De Silva H. Social cost of road crashes. Analytical presented at Australasian Transport Research Forum (ATRF), 33rd, 2010, Canberra, ACT, Australia; 2010-09, 2010; Canberra, ACT, Australia.	Not a primary study reporting VSL for Australia
43	Robinson LA, Hammitt JK. Research Synthesis and the Value per Statistical Life. *Risk Analysis: An International Journal.* 2015;35(6):1086-1100.	Not a review of VSL
44	Shah KK. Is willingness to pay higher for cancer prevention and treatment? *Journal of Cancer Policy.* 2017;11:60-64.	Not a review of VSL
45	Shah KK, Tsuchiya A, Wailoo AJ. Valuing health at the end of life: A review of stated preference studies in the social sciences literature. *Social Science & Medicine.* 2018;204:39-50.	Not a review of VSL
46	Shiftan Y, Shefer D. Evaluating the impact of transport projects: Lessons for other disciplines. *Evaluation and Program Planning.* 2009;32(4):311-314.	Not a review of VSL
47	Shmueli A, Nissan-Englcin E. Economic examination of the 2006/7 update of the Israeli national list of health services. *Harefuah.* 2008;147(6):488-492, 576.	Hebrew publication, only abstract in English

48	US Environmental Protection Agency & National Center for Environmental Economics. Valuing Mortality Risk Reductions for Environmental Policy: A White Paper. In: Agency USEP, ed. Washington DC: U.S. Environmental Protection Agency; 2010.	Unable to calculate VSL values from data reported.
49	Viscusi WK. Policy Challenges of the Heterogeneity of the Value of Statistical Life. *Foundations and Trends in Microeconomics.* 2010;6(2):99-172.	Not a review of VSL
50	Viscusi WK. The heterogeneity of the value of statistical life: Introduction and overview. *Journal of Risk and Uncertainty.* 2010;40(1):1-13.	Not a review of VSL
51	Viscusi WK. WHAT'S TO KNOW? PUZZLES IN THE LITERATURE ON THE VALUE OF STATISTICAL LIFE. *Journal of Economic Surveys.* 2012;26(5):763-768.	Not a review of VSL
52	Viscusi WK. The Value of individual and societal risks to life and health. In: Machina M, Viscusi K, eds. Vol 1: Elsevier; 2014:385-452.	Not a review of VSL
53	Viscusi WK. The Role of Publication Selection Bias in Estimates of the Value of a Statistical Life. In. Cambridge: National Bureau of Economic Research, Inc.; 2014:20116.	Working paper of a published paper included in this review
54	Viscusi WK. Pricing Lives: International Guideposts for Safety. *Economic Record.* 2018;94:1-10.	Not a review of VSL
55	Viscusi WK, Aldy JE. The Value of a Statistical Life: A Critical Review of Market Estimates throughout the World. In: Schmitz A, Zerbe RO, Jr., eds. *Applied Benefit-Cost Analysis.* Elgar Reference Collection. International Library of Critical Writings in Economics, vol. 231. Cheltenham, U.K. and Northampton, Mass.: Elgar; 2008:263-334.	Working paper originally published in 2003, reproduced in a book published in 2008
56	Viscusi WK, Masterman CJ. Income Elasticities and Global Values of a Statistical Life. *Journal of Benefit-Cost Analysis.* 2017;8(2):226-250.	Uses same dataset as a published paper included in this review
57	Wei X, Meng X. A Structural Estimation of the Employment Effects of Offshoring in the U.S. Labor Market. In. St. Louis: Federal Reserve Bank of St Louis; 2013.	Not a review of VSL (incorrect abstract in search database)

58	Wijnen W, Wesemann P, de Blaeij A. Valuation of road safety effects in cost-benefit analysis. *Evaluation And Program Planning.* 2009;32(4):326-331.	Not a review of VSL
59	Wijnen W, Weijermars W, Schoeters A, et al. An analysis of official road crash cost estimates in European countries. *Safety Science.* 2019;113:318-327.	Not a review of VSL
60	Wong B, Radin M. Benefit-Cost Analysis of a Package of Early Childhood Interventions to Improve Nutrition in Haiti. *Journal of Benefit-Cost Analysis.* 2019;10(S1):154-184.	Not a review of VSL

S6: Quality assessment of included studies

Table S6: Application of the Scale for the Assessment of Narrative Review Articles (SANRA) [1]

	1. Justification of the article's importance for the readership	2. Statement of concrete aims or formulation of questions	3. Description of the literature search	4. Referencing	5. Scientific reasoning*	6. Appropriate presentation of data∞	Total SANRA score (total of 12)
Anderson & Treich 2008 [2]	2	1	0	1	1	1	6
Bahamonde-Birke et al 2015 [3]	1	1	0	1	1	1	5
Bellavance et al 2009 [4]	2	2	2	1	1	2	10
Dekker et al 2011 [5]	2	2	2	1	1	2	10
Doucouliagos et al 2012 [6]	2	2	1	1	1	2	9
Hein et al 2016 [7]	2	2	2	1	1	2	10
Hultkrantz & Svensson 2012 [8]	2	1	2	1	2	2	10
Lindhjem et al 2011 [9]	2	2	1	1	2	2	10
Masterman & Viscusi 2018 [10]	2	1	1	1	2	2	9
Milligan et al 2014 [11]	2	2	1	1	2	1	9

Robinson & Hammitt 2016 [12]	2	2	1	2	2	1	10
Robinson et al 2019 [13]	2	1	1	1	2	2	8
Viscusi & Masterman 2017 [14]	2	1	2	1	2	2	10
Viscusi 2015 [15]	2	1	1	1	2	2	9
Wheeler & Dockins 2013 [16]	2	1	1	1	1	1	7
Yaduma et al 2013 [17]	2	2	1	2	1	2	10

*Item 5 on SANRA relates to the quality of the scientific point made in the article and reference to the quality of the evidence (e.g. randomised controlled trials, qualitative studies etc.) used to make those points. In this application to VSL review papers, the criteria relate to whether the quality of the studies impact the weight given to those studies.

∞The purpose of the VSL review studies varied (e.g. estimating publication bias, estimating benefit transfer functions etc.) and the VSL review was one component of the study. Assessment of Item 6 related to whether the data presented was appropriate given the purpose of the VSL study.

Table S7: Application of the International Society for Pharmacoeconomics and Outcome Research (ISPOR) Conjoint Analysis Checklist [18]

ISPOR Checklist	Hensher et al 2009 [19]	Hensher et al 2011 [20]
**1. Was a well-defined research question stated and is conjoint analysis an appropriate method for answering it?**	**Yes**	**Yes**
1.1 Were a well-defined research question and a testable hypothesis articulated?	The research question is: What are the WTP distributions of individuals (as car travellers) for fatality and injury avoidance?	The research question is: What are the WTP distributions of individuals (as pedestrians) for fatality and injury avoidance?
1.2 Was the study perspective described, and was the study placed in a particular decision-making or policy context?	Decision-making for road safety interventions	Given the small sample size, the authors suggest that the aim is to demonstrate how the approach can be used for valuations for road safety interventions – however recommends a larger sample size prior to applying to policy.
1.3 What is the rationale for using conjoint analysis to answer the research question?	Benefits of stated choice techniques being based on actual choice situations compared to contingent valuation which require subjects to make choices between mortality risk and money.	Same as Hensher et al 2009.
**2. Was the choice of attributes and levels supported by evidence?**	**Mostly**	**Somewhat**
2.1 Was attribute identification supported by evidence (literature reviews, focus groups, or other scientific methods)?	Attributes and levels were informed by a literature review and a pilot survey with a qualitative component.	Same as Hensher et al 2009.

2.2 Was attribute selection justified and consistent with theory?	No additional justification other than literature review and pilot survey	Same as Hensher et al 2009.
2.3 Was level selection for each attribute justified by the evidence and consistent with the study perspective and hypothesis?	Detailed justification for level selection not provided for all attributes. Levels for time and cost attributes varied +/-25% based on time and cost estimates of a recent trip as estimated by the participant.	Justification for level selection not provided.
**3. Was the construction of tasks appropriate?**	**Yes**	**Yes**
3.1 Was the number of attributes in each conjoint task justified (that is, full or partial profile)?	Full profiles are presented to all participants.	Same as Hensher et al 2009
3.2 Was the number of profiles in each conjoint task justified?	60 profiles grouped into 6 blocks so participants assess 10 profiles each. This is justified saying 'an individual cannot be expected to assess all 60 choice scenarios'.	Same as Hensher et al 2009
3.3 Was (should) an opt-out or a status-quo alternative (be) included?	For each choice situation, the respondents were allowed to choose between their initial selection and 'not to travel' in a subsequent question.	Same as Hensher et al 2009
**4. Was the choice of experimental design justified and evaluated?**	**Yes**	**Yes**

4.1 Was the choice of experimental design justified? Were alternative experimental designs considered?	Clear explanation and justification for the experimental design was provided. Three D-efficient designed were tested. The correlation of each design attribute was calculated and fixed across the blocks in order to minimise the maximum correlation. This allowed pooling of data across the 6 blocks for data analysis.	Same as Hensher et al 2009
4.2 Were the properties of the experimental design evaluated?	D-errors are reported.	D-efficient design was used.
4.3 Was the number of conjoint tasks included in the data-collection instrument appropriate?	Each of the 213 participants completed 1 task each (10 profiles).	The effective sample size was 99 with each participant completing 1 task (10 profiles)
**5. Were preferences elicited appropriately, given the research question?**	**Mostly**	**Mostly**
5.1 Was there sufficient motivation and explanation of conjoint tasks?	The survey procedure consisted of an introduction and explanation of the task, however the details of the survey script was not provided to determine whether there was sufficient motivation and explanation of the conjoint task.	Same as Hensher et al 2009.
5.2 Was an appropriate elicitation format (that is, rating, ranking, or choice) used? Did (should) the elicitation format allow for indifference?	The elicitation format was a choice between two alternatives. It did not allow for indifference – it did however allow for the choice of opting out of the preferred route in a subsequent question worded as 'if you could also choose not to travel'.	Same as Hensher et al 2009

5.3 In addition to preference elicitation, did the conjoint tasks include other qualifying questions (for example, strength of preference, confidence in response, and other methods)?	Qualifying questions related to strength of preference or confidence in response were not included. However a follow up question with an opt-out option after each of the forced choice questions was provided.	Same as Hensher et al 2009
**6. Was the data collection instrument designed appropriately?**	**Mostly**	**Yes**
6.1 Was appropriate respondent information collected (such as sociodemographic, attitudinal, health history or status, and treatment experience)?	Geographic, sociodemographic, history related to road use, attitude related to road safety and experience of road accidents information was collected for each participant.	Geographic, sociodemographic, house ownership or rental, history of pedestrian trips, experience of road accident information was collected for each participant.
6.2 Were the attributes and levels defined, and was any contextual information provided?	Survey details not provided to assess.	Contextual information provided to participants in relation to the decision context.
6.3 Was the level of burden of the data-collection instrument appropriate? Were respondents encouraged and motivated?	The surveys were completed with the assistance of a trained interviewer and therefore there were opportunities to encourage and motivate participants. The duration for the completion of the pilot survey was approximately 30 minutes, demonstrating the researchers considered the level of burden on participants.	The surveys were completed with the assistance of a trained interviewer and therefore there were opportunities to encourage and motivate participants. Consideration of level of burden not explicitly reported, however each participant only made 10 choices.

7. Was the data-collection plan appropriate?	Mostly	Somewhat
7.1 Was the sampling strategy justified (for example, sample size, stratification, and recruitment)?	Sample size was based on efficiency calculations and sampling was stratified by geographic and socioeconomic factors and recent car travel times. The final sample consisted of 12.18% of sample contacted to participate.	Sampling was stratified by geographic and sociodemographic factors. The final sample size was relatively small and inconsistently reported (sample size in tables and the text are not the same).
7.2 Was the mode of administration justified and appropriate (for example, face-to-face, pen-and-paper, web-based)?	The survey was administered face to face using a computer. This approach was appropriate and allowed participants to ask questions.	Same as Hensher et al 2009
7.3 Were ethical considerations addressed (for example, recruitment, information and/or consent, compensation)?	Recruitment was completed by a survey firm. No other details provided on ethical considerations.	Same as Hensher et al 2009
**8. Were statistical analyses and model estimations appropriate?**	**Yes**	**Yes**
8.1 Were respondent characteristics examined and tested?	Analyses to interact income and age with death and injury did not find any statistically significant effects.	Impact of sociodemographic factors on disutility estimation was tested and reported.
8.2 Was the quality of the responses examined (for example, rationality, validity, reliability)?	The rationality of the model results were reported with all parameters of the expected sign and the relative magnitude of the death and injury parameters being as would be expected.	Same as Hensher et al 2009

8.3 Was model estimation conducted appropriately? Were issues of clustering and subgroups handled appropriately?	Statistical analyses were conducted appropriately. No subgroup analyses were conducted.	Same as Hensher et al 2009
**9. Were the results and conclusions valid?**	**Mostly**	**Mostly**
9.1 Did study results reflect testable hypotheses and account for statistical uncertainty?	The results of the choice experiment are used to estimate the value of risk reduction (VRR) of urban and non-urban road accidents. Variability in the willingness to pay estimates are provided for the choice experiment, however variability around the total VVR for urban and non-urban road accidents is not provided.	The results of the choice experiment are used to estimate the value of risk reduction (VRR) of urban and non-urban pedestrian deaths and injuries. Variability in the willingness to pay estimates are provided for the choice experiment, however variability around the total VVR for urban and non-urban pedestrian accidents is not provided.
9.2 Were study conclusions supported by the evidence and compared with existing findings in the literature?	The conclusions are supported by evidence and compared to other estimates of VSL for Australia and internationally.	Same as Hensher et al 2009
9.3 Were study limitations and generalizability adequately discussed?	Limitations of the study and data used to inform the estimates were adequately covered.	Limitations of the study were adequately discussed in particular, the relatively small sample size.
**10. Was the study presentation clear, concise, and complete?**	**Yes**	**Yes**
10.1 Was study importance and research context adequately motivated?	The context and need for this research was clearly presented.	Same as Hensher et al 2009

10.2 Were the study data-collection instrument and methods described?	The data collection instrument and methods are well described, however the data collection instrument is not provided. The survey was completed on a computer, the script for the survey was not provided.	Same as Hensher et al 2009
10.3 Were the study implications clearly stated and understandable to a wide audience?	Yes	Yes

References

1. Baethge C, Goldbeck-Wood S, Mertens S. SANRA--a scale for the quality assessment of narrative review articles. Research Integrity and Peer Review. 2019;(1). doi: 10.1186/s41073-019-0064-8. PubMed PMID: edsgcl.603078578.
2. Andersson H, Treich N. The value of a statistical life. St. Louis: Federal Reserve Bank of St Louis, 2008 2008. Report No.
3. Bahamonde-Birke FJ, Kunert U, Link H. The value of a statistical life in a road safety context - A review of the current literature. Transport Reviews. 2015;35(4):488. PubMed PMID: 1688627986.
4. Bellavance F, Dionne G, Lebeau M. The value of a statistical life: A meta-analysis with a mixed effects regression model. Journal of Health Economics. 2009;28(2):444-64. doi: 10.1016/j.jhealeco.2008.10.013. PubMed PMID: 37157323.
5. Dekker T, Brouwer R, Hofkes M, Moeltner K. The effect of risk context on the value of a statistical life: A Bayesian meta-model. Environmental and Resource Economics. 2011;49(4):597-624. doi: http://link.springer.com/journal/volumesAndIssues/10640. PubMed PMID: 1253987.
6. Doucouliagos C, Stanley TD, Giles M. Are estimates of the value of a statistical life exaggerated? Journal of Health Economics. 2012;31(1):197-206. doi: 10.1016/j.jhealeco.2011.10.001. PubMed PMID: 73765886.
7. Hein L, Roberts P, Gonzalez L. Valuing a statistical life year in relation to clean air. Journal of Environmental Assessment Policy & Management. 2016;18(4):-1. doi: 10.1142/S1464333216500253. PubMed PMID: 120530328.
8. Hultkrantz L, Svensson M. The value of a statistical life in Sweden: A review of the empirical literature. Health Policy. 2012;108(2/3):302-10. doi: 10.1016/j.healthpol.2012.09.007. PubMed PMID: 83570230.

9. Lindhjem H, Navrud S, Braathen NA, Biausque V. Valuing mortality risk reductions from environmental, transport, and health policies: A global meta-analysis of stated preference studies. Risk Analysis: An International Journal. 2011;31(9):1381-407. doi: 10.1111/j.1539-6924.2011.01694.x. PubMed PMID: 66139029.
10. Masterman CJ, Kip Viscusi W. The income elasticity of global values of a statistical life: Stated preference evidence. Journal of Benefit-Cost Analysis. 2018;9(3):407-34. doi: 10.1017/bca.2018.20.
11. Milligan C, Kopp A, Dahdah S, Montufar J. Value of a statistical life in road safety: A benefit-transfer function with risk-analysis guidance based on developing country data. Accident Analysis & Prevention. 2014;71:236-47. doi: 10.1016/j.aap.2014.05.026. PubMed PMID: 97093196.
12. Robinson LA, Hammitt JK. Valuing reductions in fatal illness risks: Implications of recent research. Health Economics. 2016;25(8):1039-52. doi: 10.1002/hec.3214. PubMed PMID: 116620132. Language: English. Entry Date: In Process. Revision Date: 20160707. Publication Type: journal article. Journal Subset: Europe.
13. Robinson LA, Hammitt JK, O'Keeffe L. Valuing mortality risk reductions in global benefit-cost analysis. Journal of Benefit-Cost Analysis. 2019;(S1):15-50. Epub 01/15. doi: 10.1017/bca.2018.26.
14. Viscusi WK, Masterman C. Anchoring biases in international estimates of the value of a statistical life. Journal of Risk & Uncertainty. 2017;54(2):103-28. doi: 10.1007/s11166-017-9255-1. PubMed PMID: 123773856.
15. Viscusi WK. The role of publication selection bias in estimates of the value of a statistical life. American Journal of Health Economics. 2015;1(1):27-52. doi: http://www.mitpressjournals.org/loi/ajhe. PubMed PMID: 1503696.
16. Wheeler W, Dockins C. Meta-analysis and publication bias in the hedonic wage literature. St. Louis: Federal Reserve Bank of St Louis, 2013 2013. Report No.
17. Yaduma N, Kortelainen M, Wossink A. Estimating mortality and economic costs of particulate air pollution in developing countries: The case of Nigeria. Environmental and Resource Economics. 2013;54(3):361-87. doi: http://link.springer.com/journal/volumesAndIssues/10640. PubMed PMID: 1354228.
18. Bridges JFP, Hauber AB, Marshall D, Lloyd A, Prosser LA, Regier DA, et al. Conjoint analysis applications in health—a Checklist: A report of the ISPOR Good Research Practices for Conjoint Analysis task force. Value in Health. 2011;14(4):403-13. doi: https://doi.org/10.1016/j.jval.2010.11.013.
19. Hensher DA, Rose JM, Ortuzar JdD, Rizzi LI. Estimating the willingness to pay and value of risk reduction for car occupants in the road environment. Transportation Research: Part A: Policy and Practice. 2009;43(7):692-707. doi: http://www.sciencedirect.com/science/journal/09658564. PubMed PMID: 1059819.

20. Hensher DA, Rose JM, De Dios Ortúzar J, Rizzi LI. Estimating the value of risk reduction for pedestrians in the road environment: An exploratory analysis. J Choice Model. 2011;4(2):70-94. doi: 10.1016/S1755-5345(13)70058-7.
